# Predicting and comparing transcription start sites in single cell populations

**DOI:** 10.1371/journal.pcbi.1012878

**Published:** 2025-04-03

**Authors:** Shiwei Fu, Wei Vivian Li

**Affiliations:** 1 Department of Statistics, University of California, Riverside, Riveside, California, United States of America; The Chinese University of Hong Kong Faculty of Science, HONG KONG

## Abstract

The advent of 5’ single-cell RNA sequencing (scRNA-seq) technologies offers unique opportunities to identify and analyze transcription start sites (TSSs) at a single-cell resolution. These technologies have the potential to uncover the complexities of transcription initiation and alternative TSS usage across different cell types and conditions. Despite the emergence of computational methods designed to analyze 5’ RNA sequencing data, current methods often lack comparative evaluations in single-cell contexts and are predominantly tailored for paired-end data, neglecting the potential of single-end data. This study introduces scTSS, a computational pipeline developed to bridge this gap by accommodating both paired-end and single-end 5’ scRNA-seq data. scTSS enables joint analysis of multiple single-cell samples, starting with TSS cluster prediction and quantification, followed by differential TSS usage analysis. It employs a Binomial generalized linear mixed model to accurately and efficiently detect differential TSS usage. We demonstrate the utility of scTSS through its application in analyzing transcriptional initiation from single-cell data of two distinct diseases. The results illustrate scTSS’s ability to discern alternative TSS usage between different cell types or biological conditions and to identify cell subpopulations characterized by unique TSS-level expression profiles.

## Author summary

Gene expression is regulated not only by the total amount of RNA produced but also by where transcription begins—at transcription start sites (TSSs). Alternative TSS usage plays a critical role in cellular function and disease, but identifying and comparing TSSs at single-cell resolution remains challenging. Advances in 5’ single-cell RNA sequencing (scRNA-seq) offer new opportunities to study transcription initiation, yet current computational methods often overlook single-end data or lack robust comparative evaluations in single-cell contexts. To address this, we develop scTSS, a computational pipeline designed to predict, quantify, and compare TSS usage across single-cell populations. scTSS supports both paired-end and single-end data and integrates a statistical model to detect differential TSS usage. By applying scTSS to real data, we demonstrate its ability to uncover cell-type-specific and condition-specific TSS usage patterns, providing new insights into gene regulation at the transcriptional initiation level. Our work broadens the scope of 5’ scRNA-seq analysis and offers a valuable tool for researchers studying gene regulation in single-cell systems.

## Introduction

A transcription start site (TSS) is the specific location within a gene where RNA polymerase binds and begins transcription, marking the first base of a gene to be transcribed into an RNA transcript. In eukaryotic cells, it is possible for a gene to utilize multiple TSSs, a phenomenon called alternative transcription initiation [1,2]. It has been shown that usage of alternative TSSs is common in mammalian cells, and more than 50% of human or mouse genes have multiple TSSs [3,4]. Alternative transcription initiation contributes to the diversity of RNA isoforms, and has been shown to affect transcript stability and translation efficiency [5,6]. Moreover, alternative usage of TSSs plays an important role in transcriptional regulation and tissue-specific gene expression. For example, the Cap Analysis of Gene Expression (CAGE) data from the FANTOM consortium reveals tissue-dependent usage of alternative TSSs. The analysis of CAGE data has identified 184,476 human TSSs and 116,277 mouse TSSs across 975 human and 399 mouse samples, respectively [7,8]. Large-scale TSS switching events have also been observed during cerebellar development [9]. Besides TSS diversity in health and development, many studies have also reported alternative transcription initiation as a hallmark of disease. A large-scale analysis of RNA sequencing (RNA-seq) data from the Pan-Cancer Analysis of Whole Genomes (PCAWG), the Cancer Genome Atlas (TCGA), and the Genotype-Tissue Expression (GTEx) projects found prevalent alternative promoters associated with cancer and patient survival [10]. In particular, alternative transcription initiation is hypothesized to be a novel mechanism for oncogene activation in cancer [11].

Before the invention of single-cell RNA sequencing (scRNA-seq) technologies, computational identification and quantification of alternative transcription start sites are mostly based on data sequenced by RNA-seq or CAGE technologies [12,13]. In RNA-seq experiments, the cDNA fragments are randomly generated from the full-length transcripts, preventing deterministic identification of TSSs. In order to computationally infer TSSs, the SEASTAR method [14] uses annotation-guided transcript assembly to reconstruct the first exons of genes and quantify their usage. Another method, mountainClimber [15], predicts TSS positions based on non-uniformity of read coverage. In contrast to RNA-seq, in CAGE experiments, the cDNA fragments all originate from the 5’ of the capped transcripts, which enables direct identification of TSSs at a single-nucleotide resolution. However, identifying genuine TSSs is not trivial due to technical artifacts and stochastic transcriptional variations. One major technical artifact is the additional G-nucleotide added to the 5’ of the cDNA fragments during reverse transcription [16]. Computational methods such as CAGEr [17], TSSr [18], and TSRexploreR [19] were developed for removing artifacts and identifying genuine TSSs and TSS clusters that reflect potential promoter regions. To perform differential tests and detect usage changes in the same set of TSS clusters between different conditions, TSRexploreR utilizes DESeq2 [20] to analyze variations in TSS expression levels. In contrast, TSSr focuses on the relative usage of the TSS clusters, and uses a chi-square test to evaluate the statistical significance. However, this approach is limited to comparing only the two most highly expressed TSS clusters.

Bulk RNA-seq and CAGE technologies have allowed for the characterization of alternative TSSs in tissue and cell line samples, and with the more recent scRNA-seq technologies, there is an opportunity to study cell-to-cell variability in transcription initiation and to better understand transcriptome complexity at a single-cell resolution. There have been several library preparation methods developed to support the identification of TSSs in single cells, including C1 CAGE [21], scRCAT-seq [22], and scTSS-seq [23], which could generate short reads capturing the 5’ ends of transcripts, and ScISOr-Seq [24] and ScNaUmi-seq [25], which sequence full-length transcripts using long-read technologies. However, the limited data generated from these technologies restricts the comprehensive identification and quantification of alternative TSSs across various cell types and states. In contrast, the 10x Genomics 5’ Gene Expression assay, which has seen broader use, generates barcoded reads from the 5’ ends of transcripts. This allows for simultaneous profiling of gene expression and transcription initiation at the single-cell level. SCAFE [26] and CamoTSS [27] are two recent methods developed for TSS identification based on 5’ scRNA-seq data. SCAFE utilizes a probabilistic model based on the Poisson distribution to identify TSS clusters and incorporates a logistic regression model to remove falsely identified TSS clusters, utilizing paired ATAC-seq data when available. For instances lacking paired data, SCAFE provides a pre-trained logistic regression model within its computational package. CamoTSS employs a hierarchical clustering approach to organize identified TSSs and uses a logistic regression model similar to SCAFE’s for initial filtering. Additionally, CamoTSS enhances the accuracy of its results by integrating a convolutional neural network that assesses the sequences surrounding each cluster’s peak to further minimize false discoveries. However, both methods primarily analyze paired-end reads where only the 5’ end of Read 1 is used to precisely locate genuine TSSs. They do not explore the potential of utilizing Read 2 for approximating genuine TSS cluster locations, despite the availability of many single-end 5’ scRNA-seq datasets that could potentially be used to study TSS clusters and alternative TSS usage.

In this article, we propose a computational pipeline named scTSS that uses 5’ scRNA-seq data for predicting and comparing TSSs in single-cell populations. Our work has been partially inspired by the successes in using 3’ scRNA-seq data to model alternative polyadenylation, as demonstrated by methods such as scAPA [28], Sierra [29], scDaPars [30], MAAPER [31], and SCAPTURE [32]. For TSS prediction, we leverage the power of existing TSS prediction tools and expand the capability of scTSS by accounting for both paired-end and single-end data from multiple single-cell samples. For TSS prediction, scTSS not only utilizes existing TSS prediction tools but also enhances their capability by accommodating both paired-end and single-end data from various single-cell samples. Additionally, for differential analysis of TSS usage, we introduce a fast, easily interpretable generalized linear mixed model, which has been shown to surpass common alternatives in terms of testing accuracy or computational efficiency. Both of these functionalities are integrated into the scTSS software, providing a tailored solution for analyzing TSS usage from 5’ scRNA-seq data.

## Results

### A TSS analysis pipeline based on 5’ single-cell RNA-seq data

To facilitate the application and comparison of TSS prediction and quantification methods on 5’ scRNA-seq data, we propose a pipeline below which has been implemented in the scTSS software. The pipeline has two major steps. The first step is TSS prediction and quantification; the second step is differential TSS usage (DU) analysis. Our pipeline considers a general scenario where multiple single-cell samples (e.g., each sample is from a different donor or biological condition) are available, and a joint analysis is to be performed.

**Fig 1:**
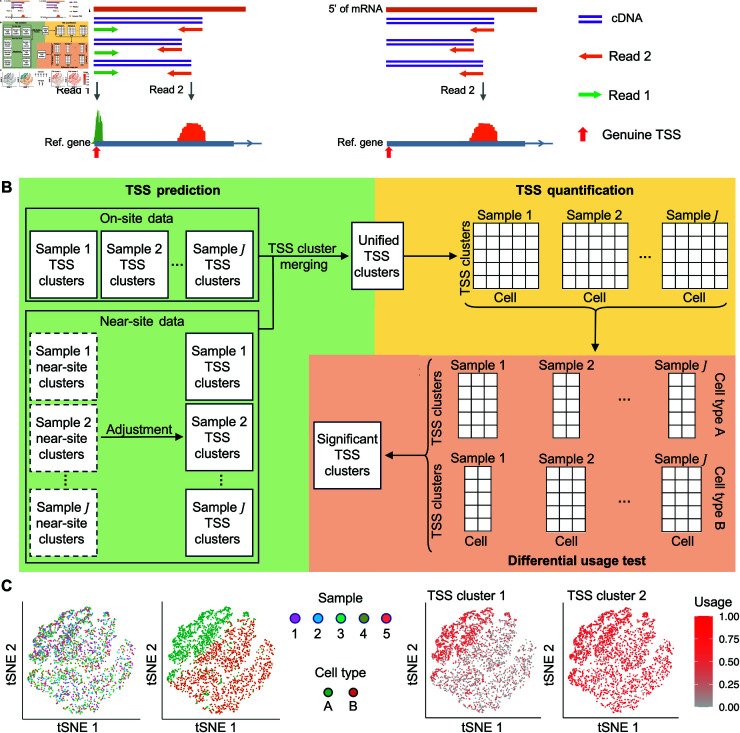
An overview of the scTSS toolkit. (A) A schematic comparison between the paired-end data (on-site data; left) and single-end data (near-site data; right). In the on-site data, Read 1 directly captures the genuine TSS. In the near-site data, Read 1 does not directly capture the genuine TSS, but there exists a predictable relationship between Read 2 and the genuine TSS, facilitating its estimation. (B) The workflow of the scTSS toolkit. scTSS consists of two major steps, TSS cluster prediction and quantification, followed by differential TSS usage (DU) analysis. In the prediction and quantification step, the toolkit takes in either predicted TSS clusters based on on-site reads, or predicted near-site clusters based on near-site reads, and eventually outputs cell-level count matrices across samples with unified TSS clusters. In the DU analysis step, we test for changes of TSS usage between biological conditions (e.g., cell types). (C) A toy example of the DU test given TSS counts from two cell types across five donors. We suppose TSS cluster 1 displays differential usage between the two cell types, with higher usage observed in cell type A, whereas the usage of TSS cluster 2 does not present significant difference between cell types. scTSS computes a *P*-value for each TSS cluster, indicating the significance of its differential usage between the two cell types.

For the first step, predictions can be performed for both individual TSSs and clusters of TSSs. In this work, we focus on the prediction of TSS clusters to facilitate multi-sample analysis and analyzing patterns of TSS distribution. Another critical consideration in TSS prediction is the type of sequencing data used. We classify 5’ scRNA-seq data into two categories: paired-end and single-end (Fig 1A). Paired-end data include cDNA information in both Read 1 and Read 2, with Read 1 being particularly useful for accurately identifying genuine TSSs. In contrast, in single-end data, only Read 2 contains cDNA information, which does not directly indicate the exact position of genuine TSSs. Read 1 only includes other information such as the cell barcode. Nonetheless, it’s feasible to infer the position of genuine TSSs based on Read 2’s position and the length of cDNA fragments. In the remainder of the manuscript, we designate the paired-end data as “on-site data” and the single-end data as “near-site data”, in order to emphasize their difference in the context of TSS prediction.

Beginning with multiple single-cell samples profiled as 5’ on-site data, the pipeline initiates with TSS prediction and clustering on individual samples using a bioinformatics tool compatible with on-site sequencing data. In the subsequent section, we compare four candidate tools. Once a set of TSS clusters is generated from each sample, our scTSS tool performs TSS cluster merging to consolidate a unified set of TSS clusters (Fig 1B; see Methods). This ensures that the TSS clusters are comparable across samples. Subsequently, read counts of the TSS clusters are summarized for each individual cell. After TSS cluster quantification, scTSS produces a count matrix of unified TSS clusters per cell for each sample.

In scenarios involving multiple single-cell samples profiled as 5’ near-site data, conventional bioinformatics tools tailored for on-site data are not directly applicable. In such cases, our pipeline initially employs a conventional tool to detect clusters of the 5’ ends of Read 2, termed as the near-site clusters (Fig 1B; see Methods). Subsequently, scTSS performs an adjustment to infer the corresponding TSS clusters from these near-site clusters. The subsequent steps in the pipeline remain consistent with those applied to the on-site data.

For the second step, the objective of DU analysis is to detect differential usage of TSS clusters associated with biological conditions, such as variations between different cell types or disparities between healthy and diseased donors (Fig 1B). TSS cluster usage is defined as the percentage of RNA transcripts originating from a gene that have a TSS within a specific cluster. To conduct DU analysis, scTSS utilizes a generalized Binomial model to assess the association between TSS cluster usage and biological conditions, while controlling for potential gene expression changes (see Methods). In an illustrative scenario depicted in Fig 1C, TSS counts from two cell types across five donors are examined. We suppose TSS cluster 1 displays differential usage between the two cell types, with higher usage observed in cell type A, whereas the usage of TSS cluster 2 remains consistent across both cell types. scTSS computes a *P*-value for each TSS cluster, indicating the significance of its difference between the two cell types.

### Accuracy of TSS prediction on 5’ single-cell on-site data

For the TSS prediction task, we considered four existing tools developed for on-site data by incorporating them into the scTSS pipeline (see Methods). Three of these tools were originally designed for CAGE-seq data (CAGEr [17], TSSr [18], and TSRexploreR [19]), and one tool was specifically designed for 5’ scRNA-seq on-site data (SCAFE [26]). A detailed feature comparison between these methods is summarized in S1 Table. For the numerical comparison, we used a COVID-19 (paired-end; on-site) dataset generated from the 10x Genomics Single Cell 5’ Library [33]. This dataset contains 22 single-cell samples of peripheral blood mononuclear cells (PBMCs) from 13 different donors, including 5 samples from healthy donors, 4 samples from COVID-19 patients with severe symptoms, 7 samples from COVID-19 patients with moderate symptoms, and 6 samples from convalescent patients.

**Fig 2:**
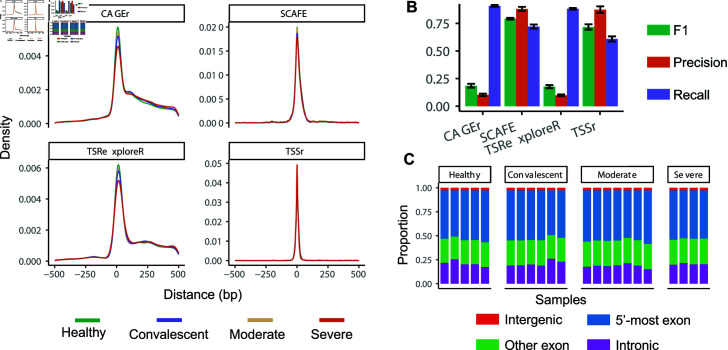
TSS prediction results of the COVID-19 dataset. (A) The distribution of genomic distance between predicted dominant TSSs and the corresponding annotated TSSs using all genes with one annotated TSS. A positive distance means the predicted dominant TSS is on the 5’ side of the annotated TSS and vice versa. The analysis was restricted to TSS clusters whose predicted dominant TSSs fell within 500 bp of the annotated TSSs, since a larger distance more likely indicates missing annotations. (B) Accuracy of TSS cluster prediction compared with the complete FANTOM5 annotation. The precision, recall, and F1 scores were first calculated for each sample, and then averaged across samples. The error bars indicate the standard deviation of the scores. (C) The proportion of different types of predicted dominant TSSs based on SCAFE. The classification of TSS locations was based on the GRCh38 reference.

After applying the above four methods to the COVID-19 dataset via our scTSS pipeline, we first compared their accuracy in TSS cluster prediction. To assess the accuracy of predicted TSS clusters, we compared them with annotated TSSs from the FANTOM5 dataset [34]. It is important to note that the FANTOM5 annotation encompasses samples from various tissue and cell types, whereas the COVID-19 dataset exclusively consists of PBMCs. To ensure a more reliable evaluation, we retained an annotated TSS if it was detected in more than 30 FANTOM5 samples of immune cells. Using the FANTOM5 annotation as a reference, we computed the genomic distances between the dominant TSS (the most frequently used TSS within a TSS cluster) in each predicted TSS cluster and the closest annotated TSS, particularly focusing on genes with only one annotated TSS (Fig 2A). Analyzing the distributions of these distances, we observed a center around 0 bp regardless of the TSS prediction method being used, suggesting consistent agreement between the predicted TSS clusters and annotated TSSs. In addition, it’s worth noting that the standard deviation of distance was 46 bp and 85 bp for TSSr and SCAFE, respectively, significantly smaller than those observed for CAGEr (181 bp) and TSRexploreR (183 bp).

Next, we assessed the accuracy of TSS prediction by calculating the precision, recall, and F1 scores of the four methods based on the complete set of annotated TSSs in the FANTOM5 project (Fig 2B; Methods). CAGEr and TSRexploreR showed higher recall rates but lower precision compared to TSSr and SCAFE, resulting in lower overall F1 scores. This result suggests that CAGEr and TSRexploreR may be more prone to false positive predictions on single-cell data. We also calculated accuracy focusing on genes with only one annotated TSS in FANTOM5 (S1 Fig). Consistent with our previous observations based on the all-genes evaluation, SCAFE still achieved the highest F1 score among all methods, followed by TSSr. The high precision of SCAFE may be attributed to the fact that it utilizes a logistic regression model to filter genuine TSSs based on extracted TSS cluster features. We also evaluated the nucleotide proportions at the -1 and +1 positions of the dominant TSSs for each method (S1 Fig). Previous studies suggest a strong preference for PyPu nucleotide (a pyrimidine followed by a purine) at these positions [35]. Notably, both SCAFE and TSSr exhibited higher PyPu proportions at -1 and +1 positions of their predicted dominant TSSs, suggesting they are more precise in predicting TSSs from single-cell on-site data.

We then summarized the statistics of predicted TSS clusters based on the results of SCAFE. On average, SCAFE identified 27,163 TSS clusters from 14,481 genes in each single-cell sample, with a standard deviation of 2,819. An average of 5,494 genes had multiple predicted TSS clusters (S1 Fig), and the average width of the TSS clusters for each sample ranged from 76 bp to 83 bp (S1 Fig). Furthermore, we studied the categories of the TSS clusters based on the genomic locations of their dominant TSSs in the GRCh38 reference (Fig 2C). We observed that the proportions of dominants TSSs located in the 5’-most exons versus other exons were consistent across all samples, irrespective of the disease condition. Then, we also quantified the relative usage of TSS clusters in each category based on the number of reads mapped to them (S1 Fig). The abundance of intronic TSS clusters tended to be higher in healthy samples compared to samples under other conditions. This observation suggests that alternative TSS usage may contribute to the response to disease conditions.

### Feasibility of TSS prediction on 5’ single-cell near-site data

To predict TSS clusters from 5’ single-cell near-site data, we treated Read 2 from the COVID-19 dataset as the near-site data by disregarding the availability of Read 1. We included CAGEr, TSSr, and TSRexploreR in our analysis by accounting for the near-site data through the scTSS pipeline (Fig 1A; see Methods). SCAFE was not considered as it is not applicable to near-site data. First, we assessed the distribution of the genomic distance between predicted TSS cluster centers and the corresponding annotated TSSs using genes with one annotated TSS in FANTOM5 (S2 Fig). Compared with the on-site data analysis using Read 1 of the same dataset, we still observed the centering around 0 bp with all three methods, with TSSr leading to the most noticeable peak. The standard deviation of the distances for TSSr, CAGEr, and TSRexploreR was 146 bp, 204 bp, and 185 bp, respectively. Second, we calculated prediction accuracy scores using all genes in the FANTOM5 annotation as a reference (S2 Fig). Although TSSr achieved the highest F1 score, its accuracy was significantly lower than that achieved on the on-site data.

To further compare TSS predictions from on-site and near-site data derived from the COVID-19 dataset, we calculated sample-wise accuracy scores for predictions on the near-site data, using predictions on the on-site data as the reference (S2 Fig). This analysis revealed relatively high precision but low recall rates, indicating that most TSS clusters identified from the near-site data (Read 2) were also identified from the on-site data (Read 1). The lower recall rate was likely due to the smaller number of TSS clusters identified from near-site data: using TSSr, we predicted an average of 16,235 TSS clusters per sample from on-site data, compared to 7,567 TSS clusters per sample from near-site data. These results suggest that adapting the TSSr method within the scTSS pipeline for predicting TSS clusters from 5’ single-cell near-site data analysis is feasible, although it yields lower overall accuracy compared to on-site data. Lastly, we summarized the proportions of different categories of TSS clusters based on their center positions (S2 Fig). When compared to TSS clusters identified from the on-site data, we observed an increase in the proportion of clusters in the “other exon” and “intergenic” categories in the near-site data. This discrepancy is likely attributable to the exclusion of near-site clusters originating in annotated intronic regions, as required by the adjustment approach.

**Fig 3:**
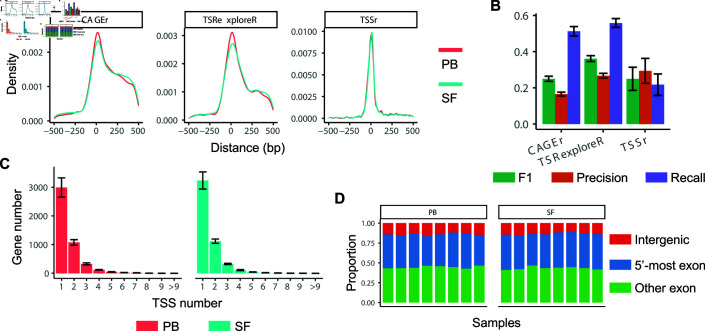
TSS prediction results of the Arthritis dataset. (A) The distribution of the genomic distance between predicted TSS cluster centers and the corresponding annotated TSSs using all genes with one annotated TSS. A positive distance means the predicted center is on the 5’ side of the annotated TSS and vice versa. The analysis was restricted to TSS clusters whose centers fell within 500 bp of the annotated TSSs. (B) Accuracy of the TSS cluster prediction compared with the FANTOM5 annotation. The precision, recall, and F1 scores were first calculated for each sample, and then averaged across samples. The error bars indicate the standard deviation of the scores. (C) Gene numbers with varying number of predicted TSS clusters based on the prediction of TSSr. The bars represent the mean value across samples, and the error bars represent the the standard deviation. (D) The proportion of different types of predicted TSS cluster centers based on TSSr. The classification of center locations was based on the GRCh38 reference. When the original near-site clusters were located in an intronic region, indicating missing annotations, we could not confirm the locations of genuine TSS centers. Therefore, we excluded those clusters from the categorization.

Next, we applied the scTSS pipeline (with CAGEr, TSSr, or TSRexploreR) to a single-end (near-site) dataset generated from the 10x Genomics Single Cell 5’ Library [36]. This dataset includes 16 single-cell samples of either synovial fluid (SF) or peripheral blood (PB) from eight arthritis patients, with one SF sample and one PB sample from each patient. We refer to this dataset as the Arthritis dataset. We first evaluated the accuracy of TSS clustering based on the distance between the center of the predicted TSS clusters and the annotated TSSs for genes with a single annotated TSS (Fig 3A). The same FANTOM5 annotation as in the COVID-19 on-site data analysis was used. We still observed the centering around 0 bp with all three methods, with TSSr led to the most noticeable peak. Nevertheless, compared to the on-site data, predictions on near-site data resulted in higher variability. The standard deviation of the distances for TSSr, CAGEr, and TSRexploreR was 132 bp, 205 bp, and 194 bp, respectively. We further evaluated the accuracy of TSS clustering using all genes based on the precision, recall, and F1 scores (Fig 3B), and observed a similar pattern as in the on-site data analysis. Compared with CAGEr and TSRexploreR, TSSr had higher precision but lower recall rates. When restricting to one-annotated-TSS genes, TSSr outperformed the other two methods in both precision and recall (S3 Fig).

Based on the near-site clusters generated by TSSr, the scTSS pipeline identified an average of 7,378 TSS clusters from 4,765 genes per single-cell sample, with a standard deviation of 510. In addition, an average of 1,650 genes had multiple predicted TSS clusters (Fig 3C). The average length of these predicted TSS clusters ranged from 130 bp to 142 bp (S3 Fig). For the Arthritis dataset, we also summarized the categories of TSS clusters based on the genomic locations of their centers (Fig 3D). In both PB and SF samples, the majority of the cluster centers were located on non-5’-most exons, followed by 5’-most exons and intergenic regions. We then assessed the average usage of different categories of TSS clusters, which revealed that approximately 50% of RNA transcripts originated from the 5’-most exons, while the remaining 50% originated from other exons (S3 Fig).

### Comparison of DU analysis based on simulated single-cell data

After TSS clusters are predicted and quantified, an important task is to perform differential usage (DU) analysis to identify TSS clusters whose relative usage significantly differs between biological groups. There have been methods developed for differential usage of transcripts based on bulk sequencing data, such as DEXSeq [37], which can be applied to perform DU analysis on pseudo-bulk data transformed from single-cell samples. There have also been methods for differential gene expression analysis, such as MAST [38], IDEAS [39], and the Wilcoxon rank sum test (both at single-cell and pseudo-bulk levels), which may be adapted for DU analysis (see Methods). However, their actual performance in the context of DU analysis given single-cell samples from multiple donors has not been comprehensively evaluated. To evaluate the accuracy of these methods in DU analysis, we designed a simulation study considering sample-level variability with different sample sizes. In this study, we also considered two Binomial generalized linear mixed models (GLMMs): one at the single-cell level and the other at the pseudo-bulk level. The Binomial GLMM is capable of modeling gene expression changes as a confounding factor while simultaneously accounting for sample-level variability (see Methods).

In the simulation, we considered the differential TSS usage analysis between two biological groups (i.e., two cell types). We generated TSS-cluster-level expression data corresponding to different number of single-cell samples per group (5, 10, 15, 20, and 25), combined with varying cell numbers per sample (100 and 500). In each single-cell sample, there were 2,000 genes. Of these, 1,000 genes had TSS clusters following the null hypothesis (same mean usage in the two cell types), while the other 1,000 genes had TSS clusters following the alternative hypothesis (different mean usage in the two cell types). In addition to these simulation settings, we considered the existence of outliers and evaluated method performance given different degrees of outlier frequency (represented by *π*) and outlier deviation (see Methods). After applying the aforementioned methods for DU analysis, we calculated their type I errors and statistical power, using a threshold of 0.05 on *P*-values corrected by the Benjamini-Hochberg procedure.

**Fig 4:**
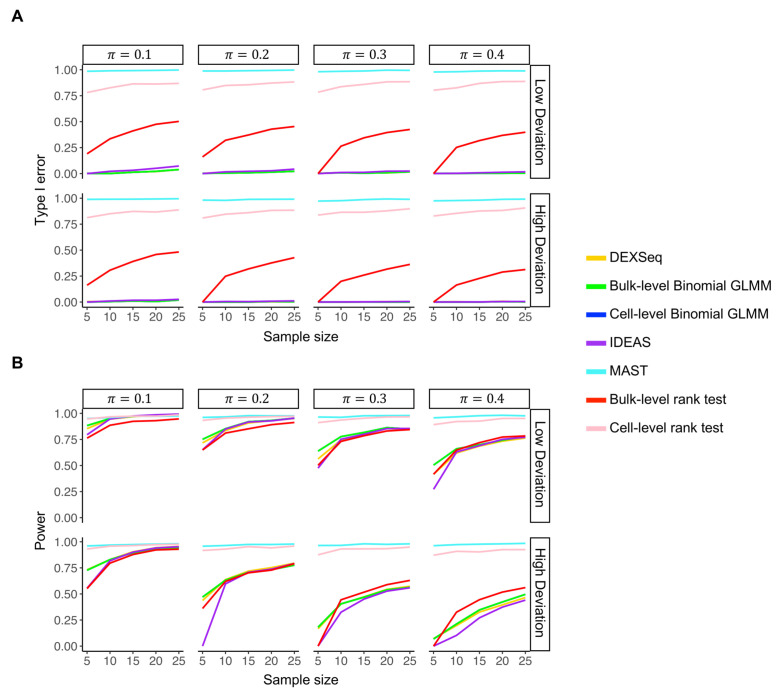
Simulation study of DU analysis with 500 cells per sample. (A) Comparison of type I error rates between seven TSS DU testing methods given various sample size, outlier frequency (*π*), and degree of outlier deviation. (B) Comparison of statistical power between the seven TSS DU testing methods. The results of bulk-level and cell-level Binomial GLMMs were virtually identical, resulting in overlapping lines.

In Fig 4, we present the simulation results when the number of cells in each sample was 500. The results show that MAST and the Wilcoxon test (both at single-cell and pseudo-bulk levels) were not able to control the type I error at the target level (Fig 4A). We observed a type I error rate close to 1 for MAST in all simulation settings. Since MAST directly models read counts, it cannot account for potential gene expression changes between biological groups, which lead to changes in observed TSS-cluster-level counts even when TSS usage remains constant. While the Wilcoxon test had a smaller type I error rate than MAST, its error was still highly inflated in most settings. Furthermore, the single-cell-level Wilcoxon test experienced more severe type I error inflation compared to the pseudo-bulk-level test. In contrast, DEXSeq, IDEAS (modified for DU analysis), and Binomial GLMMs demonstrated effective type I error control. For Binomial GLMM, the single-cell-level and pseudo-bulk-level models led to almost identical results, so the two lines overlap in Fig 4. When the number of cells per sample was reduced to 100 (S4 Fig), we obtained similar results, with DEXSeq, IDEAS, and Binomial GLMM more effectively controlling the type I error compared with MAST and the Wilcoxon tests.

Regarding statistical power, we found that the Binomial GLMM and DEXSeq, both parametric models, demonstrated better statistical power than IDEAS, a nonparametric model, particularly when the number of samples was small or the outlier deviation was high. This observation held constant across different cell counts per sample (Fig 4B and S4 Fig). In most simulation settings, the statistical power of the Binomial GLMM and DEXSeq was highly comparable, with DEXSeq showing slightly lower power under conditions of small sample size or high outlier deviation. Using the Binomial GLMMs, we observed again that the tests at the single-cell and pseudo-bulk levels yielded almost identical results, with the difference in *P*-values for each TSS cluster being less than $10^{-4}$. In addition, we observed the expected trend of decreasing power with increasing outlier frequency or deviation for all three models.

We also compared the running time of these methods (S5 Fig). The pseudo-bulk level Binomial GLMM was the fastest method when the cell number per sample was 500, followed by DEXSeq and the pseudo-bulk-level rank test, all of which demonstrated rapid execution speeds. In summary, our simulation analysis identifies pseudo-bulk-level Binomial GLMM and DEXSeq as the two best methods for DU analysis for their higher accuracy, robustness to outliers, and computational efficiency.

### scTSS reveals differential TSS usage in real data

After evaluating the accuracy of DU analysis on simulated data, we employed the scTSS pipeline to identify differential TSS usage between B cells and activated T cells from the COVID-19 dataset, using samples from healthy donors. On average, there were 579 activated T cells and 387 B cells in each sample. We used SCAFE for the prediction of TSS clusters, and the pseudo-bulk-level Binomial GLMM for differential testing. In the DU analysis, we filtered out genes expressed in fewer than 10% cells (combining activated T and B cells) per sample and genes with only a single identified TSS cluster. After the filtering step, we performed the differential TSS usage test on 3,243 TSS clusters in 1,397 genes, and obtained 981 differential TSS clusters with BH-adjusted *P*-values  <  0.05.

**Fig 5:**
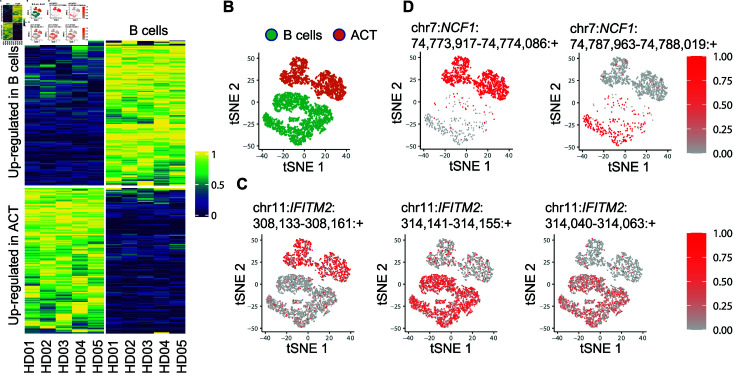
Differential TSS usage analysis between the activated T (ACT) cells and B cells in the COVID-19 dataset. (A) Heatmap of normalized average TSS usage in ACT and B cells. For each TSS cluster, its sample-specific usage was first calculated by taking the average across all cells in that sample. Then, the average usage was normalized across samples using the min-max normalization. (B) tSNE plot of all ACT and B cells among healthy donors. (C) tSNE plots of ACT and B cells colored by the usage of different TSS clusters on *IFITM2*. Cells in which *IFITM2* was not detected were excluded. (D) tSNE plots of ACT and B cells colored by the usage of different TSS clusters on *NCF1*. Cells in which *NCF1* was not detected were excluded.

Based on the *P*-values yielded by scTSS, we selected the top 200 TSS clusters up-regulated in each cell type, and compared their average usage in different samples. A clear pattern of differential TSS usage can be observed between B cells and activated T cells (Fig 5A). For example, three TSS clusters were identified on the *IFITM2* gene, and all of them presented obvious differential usage between the two cell types (BH-adjusted *P*-values  <  $10^{-5}$). Among them, one TSS cluster (chr11:308,133-308,161:+) was up-regulated in activated T cells and the other two (chr11:314,141-314,155:+ and chr11:314,040-314,063:+) were up-regulated in B cells (Fig 5B-C). This new observation is complementary to the previous finding that the expression of *IFITM2* is up-regulated during T-cell activation [40]. Another example is the *NCF1* gene, on which we identified two TSS clusters, and both of them demonstrated differential usage (*P*-values  <  $10^{-5}$) (Fig 5D). *NCF1* encodes neutrophil cytosolic factor 1, which was shown to regulate T cell activation in autoimmunce diseases like arthritis [41]. To further analyze the top differentially used TSSs identified by scTSS, we conducted a motif analysis on their promoter sequences (S6 Fig; Methods). The motifs with the smallest *P*-values included those of ETS1 and RUNX1, which have been reported as important factors in the development of T cell lineages [42,43], as well as motifs of ERG and PU.1, which are responsible for controlling B cell programs [44,45]. It is also worth noting that, based on a differential gene expression (DGE) analysis of all 1,397 genes used in the DU analysis, differences in TSS usage may arise from regulatory mechanisms independent of gene expression changes. Among the genes analyzed, 37.3% were not differentially expressed but exhibited differentially used TSS clusters, while 9.81% were differentially expressed without corresponding changes in TSS cluster usage (S7 Fig).

Since DEXSeq and Binomial GLMM demonstrated similar performance in the simulation study, we also compared their effectiveness in detecting differentially used TSS clusters between activated T cells and B cells. The comparison suggested a strong agreement between the two sets of identified differential TSS clusters, with 835 TSS clusters reported by both methods (S8 Fig). Another 146 and 25 TSS clusters were uniquely identified by Binomial GLMM and DEXSeq, respectively. We ranked the top 200 differentially used TSS clusters based on the *P*-values generated by Binomial GLMM and compared their ranks as determined by DEXSeq (S8 Fig). Our analysis revealed a high level of agreement between the two methods. Notably, for the top 115 TSS clusters, there was complete concurrence in their rankings between these methods.

Next, we employed the scTSS pipeline to identify differential TSS usage between T cells from the SF and PB samples in the Arthritis dataset. We used TSSr for the prediction of near-site TSS clusters, and pseudo-bulk-level Binomial GLMM for differential testing. After we filtered out genes expressed in fewer than 50% samples and genes with only a single predicted TSS cluster, the differential test was performed on 9,060 TSS clusters from 3,019 genes, and led to 74 differential TSS clusters with BH-adjusted *P*-values  <  0.05. The usage of the 40 most significant TSS clusters are displayed in S9 Fig. A clear pattern of differential TSS usage between the PB and SF samples can be observed. For example, a TSS cluster on the *CHCHD5* gene (chr2:112,585,869-112,585,937:+) presented higher abundance in PB samples compared with SF samples across the eight patients (S9 Fig). In contrast, another TSS cluster on the *UBR2* gene (chr6:42,563,957-42,564,104:+) demonstrated higher abundance in SF samples than PB samples. These two TSS clusters were both among the 10 most significant TSS clusters identified by the Binomial GLMM. Similar to the observation from the COVID-19 analysis, we observed little overlap between genes with differential expression and differential TSS usage in the Arthritis data analysis (S7 Fig).

We also compared the differential testing results of the Binomial GLMM and DEXSeq on this dataset. The two methods identified 31 common differential TSS clusters, while DEXSeq and Binomial GLMM uniquely identified 6 and 43 differential TSS clusters, respectively (S10 Fig). Furthermore, we ranked the top 50 differentially used TSS clusters based on the *P*-values generated by Binomial GLMM and compared these ranks with those determined by DEXSeq (S10 Fig). Similar to the COVID-19 data analysis, we observed a high level of concordance between the two methods among the most significant TSS clusters. We then closely examined the differential TSS clusters identified exclusively by each method. For Binomial GLMM, 19 of the unique TSS clusters were up-regulated in SF and the other 24 TSS clusters were down-regulated in PB (S11 Fig). The two most significant TSS clusters were chr16:29,806,824-29,806,868:+ (on gene *MAZ*) and chr1:28,369,595-28,369,608:+ (on gene *PHACTR4*), both showing higher usage in the PB samples (S12 Fig). For DEXSeq, all 6 TSS clusters uniquely identified by it were up-regulated in PB (S11 Fig). Among these, the top two TSS clusters were chr2:88,857,651-88,857,685:- (on gene *IGKC*) and chr14:9,002,994-9,003,015:+ (on gene *ERI1*) (S13 Fig).

To further evaluate the performance of the bulk-level Binomial GLMM and DEXSeq, we conducted additional sub-sampling analyses using both the COVID-19 and Arthritis datasets. For each dataset, two types of sub-sampling were performed: (1) sub-sampling cells within each sample and (2) sub-sampling samples within each condition. In the cell-level sub-sampling, we randomly selected 80%, 60%, and 40% of cells from each sample and conducted DU testing using both methods. In the sample-level sub-sampling, we randomly selected subsets of samples within each condition for DU testing. A TSS cluster was considered differentially used if its BH corrected *P*-value was  <  0.05. Each sub-sampling procedure was repeated 10 times. In the sub-sampling analysis of the COVID-19 dataset (S14 Fig), consistent with the findings from the original data, we observed a significant overlap between the differential TSS clusters identified by the two methods. However, the bulk-level Binomial GLMM consistently identified more differential TSS clusters than DEXSeq in both cell-level and sample-level sub-sampling analyses, suggesting superior statistical power. Similarly, in the sub-sampling analysis of the Arthritis dataset (S14 Fig), across most parameter settings, the bulk-level Binomial GLMM consistently identified a greater number of differential TSS clusters compared to DEXSeq, with the majority of TSS clusters identified by DEXSeq also detected by the bulk-level Binomial GLMM. In the small sample size scenario with only three samples in each condition, DEXSeq outperformed bulk-level Binomial GLMM but its performance was very unstable across independently repeated experiments.

### scTSS enables discovery of cell subpopulations based on TSS-specific expression

**Fig 6:**
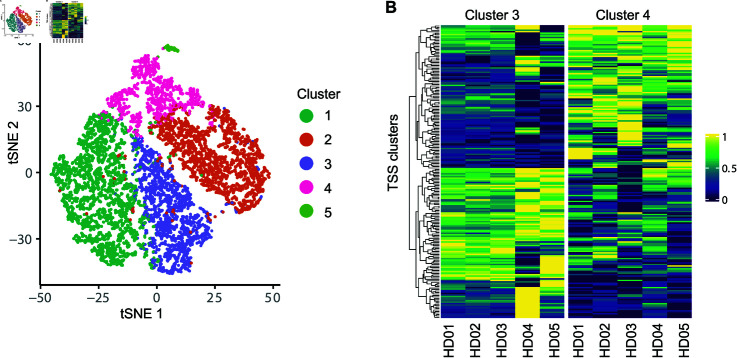
Clustering analysis of naïve T cells based on TSS-specific expression. (A) tSNE plot of naïve T cells based on TSS-specific expression. (B) Heatmap of sample-level TSS usage between clusters 3 and 4. The top 200 differential TSS clusters were selected based on *P*-values. For each TSS cluster, the min-max normalization was performed on its usage across samples.

As TSS isoforms have been shown to present cell-type-specific expression, we next investigated the possibility to identify cell subpopulations using the abundance of TSS clusters. For this analysis, we selected all the naïve T cells from the five healthy donors in the COVID-19 dataset. On average, there were 1,629 naïve T cells per sample with a standard deviation of 155. After obtaining the TSS usage matrices of these samples, we performed a clustering analysis based on TSS usage (see Methods), and identified five clusters in the naïve T cells (Fig 6A).

To further evaluate the difference in TSS cluster usage between the five identified clusters, we applied scTSS (bulk-level Binomial GLMM) to perform the DU test. We focused on cluster 3 and cluster 4, both of which include at least 50 cells from each sample. After we filtered out genes expressed in fewer than 10% cells (combining cells in cluster 3 and 4), 3,390 TSS clusters were tested, and 2,034 of them had BH-adjusted *P*-values  <  0.05. We selected the top 200 TSS clusters based on *P*-values and visualized their relative usage by sample and cluster (Fig 6B). Hierarchical clustering of the TSS clusters revealed two distinct patterns: one group of 105 TSS clusters had higher abundance in cluster 3, and the other group of 95 TSS clusters had higher abundance in cluster 4. This observed pattern was consistent among the five donors. Our analysis demonstrates that usage of TSS clusters can be used to identify cell subpopulations with distinct TSS expression patterns.

## Discussion

In this article, we introduce the scTSS pipeline, which offers two primary functionalities: TSS prediction and quantification using 5’ scRNA-seq data, and differential TSS usage analysis across different cell types or biological conditions. For TSS prediction with on-site data, scTSS leverages existing bioinformatics tools, whose performance we have benchmarked against FANTOM5 TSS annotations. For near-site data, where no compatible tools currently exist, we have developed an adjustment procedure that refines near-site clusters to approximate genuine TSS locations. Regarding TSS quantification, scTSS provides a user-friendly workflow that generates a cell-level TSS count matrix for each sample. For differential analysis, scTSS employs a generalized binomial model to assess the association between TSS cluster usage and biological conditions, while controlling for potential gene expression changes and sample-level TSS usage variability. Our results demonstrate that differential TSS usage analysis can reveal variations in TSS usage between biological conditions, which are complementary to gene-level expression changes. Furthermore, alternative TSS usage can identify subpopulations of single cells characterized by distinct, TSS-specific expression patterns.

We would like to discuss several limitations and potential future directions for this work. First, while we have compared the predicted TSSs with those annotated in FANTOM5, it is important to recognize that these annotations should not be treated as the definitive ground truth for expressed TSSs in single-cell samples. Technologies specifically designed to capture the 5’ ends of mRNA transcripts, such as those reported in [21–23], will be crucial in future studies. Generating paired data from these technologies alongside the 10x Genomics 5’ Gene Expression assays will provide complementary data that can further validate the TSS predictions in single-cell contexts. Second, we have shown that adjusting near-site clusters allows for the prediction of TSS clusters, yet this adjustment predominantly relies on existing exonic annotations, which lacks comprehensiveness and cell-type specificity. Currently, this method does not accommodate near-site clusters located in annotated intronic regions, potentially diminishing the accuracy of TSS cluster predictions relative to on-site data. As demonstrated by the paired comparison between on-site and near-site data analyses based on the same COVID-19 dataset, the TSS clusters identified from near-site data generally exhibit higher variability and a lower recall rate compared to those identified from on-site data. While the relatively high precision highlights the potential of near-site data analysis for uncovering biological insights, the limited recall hinders a comprehensive understanding of alternative TSS usage. To enhance the method’s effectiveness, a potential extension is to incorporate a more sophisticated probabilistic model for TSS inference, similar to those employed in RNA isoform reconstruction and alternative polyadenylation identification [31,46]. Third, we have presented the clustering analysis of naive T cells as an example to demonstrate the potential for identifying cell subpopulations using TSSs predicted and quantified by scTSS. Subsequent to this clustering, we conducted a differential usage test to compare TSS usage between clusters 3 and 4. It is important to note that this DU test is intended as an exploratory tool to generate hypotheses rather than a robust method for post-selection inference. Similarly, performing differential gene expression analysis after clustering with the same data is subject to analogous limitations. A recent method that employs count splitting to overcome this issue [47] could potentially be adapted for identifying differentially used TSSs in post-clustering analysis of single-cell data. Lastly, we would like to acknowledge the challenges posed by low gene expression levels in both TSS identification and DU analysis. For genes with low expression, TSS identification becomes more difficult, leading to a lower recall rate for TSSs located on these genes. Moreover, even when TSSs for these genes are successfully identified, the limited read coverage makes it challenging to accurately estimate their relative usage in single cells. To mitigate this issue, we applied gene filtering based on their detection rates in both simulation and real data studies. This filtering approach allowed us to focus on genes with sufficient expression levels, thereby improving the reliability and accuracy of our TSS identification and DU analyses.

In summary, the scTSS pipeline provides computational methods that facilitate the measurement and comparison of TSS-specific gene expression for both on-site and near-site single-cell data. Although our primary focus has been on data from the 10x Genomics 5’ Gene Expression Assay, which is the most widely used technology for generating 5’ scRNA-seq data, scTSS is also applicable to other technologies that produce data in a similar format. We anticipate that scTSS will be a valuable tool for investigating transcriptional regulation and cell-type-specific alternative TSS usage within single-cell populations.

## Methods

### TSS cluster prediction for a single sample

The first step in the scTSS pipeline is to perform TSS cluster prediction on individual single-cell samples. By applying a TSS cluster prediction tool through the scTSS pipeline, we aim to obtain a set of identified TSS clusters for each single-cell sample. We categorize 5’ 10x Genomics Gene Expression Assay into two types: paired-end (on-site) and single-end (near-site) (Fig 1A). In on-site data, both Read 1 and Read 2 contain cDNA information. Significantly, Read 1 can be used to accurately identify the true TSSs, enabling the application of TSS cluster prediction tools originally designed for CAGE-seq data to predict TSS clusters in this type of sequencing. In contrast, in near-site data, only Read 2 carries cDNA information and does not directly pinpoint the exact location of the authentic TSSs. Nonetheless, the gap between the 5’ end of Read 2 and the actual TSS depends on the length of the cDNA fragment.

In the context of on-site data, a variety of TSS cluster prediction tools, originally developed for CAGE-seq data, can be utilized. Examples include CAGEr [17], TSSr [18], and TSRexploreR [19]. These methods are all designed to remove any potential additional G-cap at the 5’ end of Read 1, thereby enabling precise identification of TSSs. However, a common challenge we have observed is memory exhaustion when handling large BAM files, which is common in single-cell data analysis. To mitigate this in the scTSS pipeline, we first convert the BAM file into a mapped TSS table before performing TSS cluster analysis. The mapped TSS refers to a genomic location identified by mapping the 5’ end of Read 1 to the genome. A mapped TSS table is a tab-separated file including the following columns: chromosome, location, count, and strand. Using the mapped TSS table instead of the BAM file can effectively avoid memory exhaustion while retaining the necessary information needed to identify TSS clusters. Notably, two recent tools named SCAFE [26] and CamoTSS [27] are specifically tailored for single-cell on-site data, offering enhanced TSS clustering with potential removal of strand invasion artifacts. In situations where these tools, specifically for those designed for bulk CAGE-seq data, are not able to yield single-cell level expression matrices, scTSS incorporates a solution, enabling the generation of single-cell TSS cluster expressions, which will be discussed in subsequent sections.

For near-site data, scTSS provides a method to estimate the locations of genuine TSS clusters. The method begins by obtaining the locations of near-site clusters, which are identified from the 5’ end of Read 2, using a TSS cluster prediction tool for on-site data. After obtaining the near-site TSS clusters, we adjust their locations to approximate genuine TSS clusters. First, when the original near-site clusters are located in an intronic region, indicating missing annotations, we exclude them from downstream analysis as it is not possible to determine the genuine TSSs. Next, we learn the distribution of the distance between the near-site clusters and corresponding genuine TSSs using genes with a single annotated TSS. For each gene, we calculate the smallest distance between the centers of near-site TSS clusters and the annotated TSS. Distances greater than 1,000 bp are ignored, as they most likely indicate the presence of novel, unannotated TSSs. As an example, the distributions of the Arthritis dataset are shwon in S3 Fig. The weighted mean of all distances is then calculated across the single-annotated-TSS genes, where the weight corresponds to the read number supporting the near-site clusters. This weighted mean is referred to as the adjustment distance. Finally, we adjust the near-site clusters by this adjustment distance to obtain the estimated TSS clusters. The adjustment is performed on the transcriptome to ensure the procedure is intron-aware.

### Differential TSS cluster usage analysis between cell populations

**(1) TSS cluster merging for multiple samples**

When TSS cluster prediction tools analyze samples individually, directly comparing TSS clusters across different samples becomes challenging due to the prediction of non-identical but overlapping clusters. An example of this challenge is when the predicted TSS clusters for the same gene show slight variations in their positions but correspond to the same set of genuine TSS isoforms. Therefore, when our objective involves comparing the utilization of TSS clusters among different samples, it becomes necessary to carry out TSS cluster merging beforehand. To achieve the merging of TSS clusters across various samples, scTSS first pools predicted TSS clusters from all samples. Following this, the pooled TSS clusters are separated into unified, non-overlapping TSS clusters using the “disjoin" function from the R package GenomicRanges [48]. Then, we can perform TSS cluster quantification in each sample for a unified set of TSS clusters.

**(2) TSS cluster quantification at single-cell level**

For on-site data, the expression level of a TSS cluster within an individual cell is determined by counting the reads from that cell whose 5’ end of Read 1 is located within the TSS cluster. For near-site data, the expression level of a TSS cluster is derived from its corresponding near-site cluster, by counting Read 2 whose 5’ end is located within the near-site cluster from that cell.

**(3) Differential usage analysis by generalized linear mixed models**

In this section, we introduce the binomial generalized linear mixed model (GLMM) used in the differential TSS usage analysis, which is also incorporated in the scTSS pipeline and software. We quantify the observed usage of a TSS cluster as the proportion of transcripts from a gene using any TSS in this cluster. For example, for a gene with *T* TSS clusters, the observed usage of TSS cluster *t* ( *t* = 1 , 2 , *…* , *T* )  is defined as:


$$\begin{array}{ccc} \theta_{t}=x_{t}∕\overset{T}{\underset{t=1}{\sum }}x_{t}\;, & & \end{array}$$ (1)

where $x_{t}$ is the transcript count of the *t*-th TSS cluster. In the context of single-cell data with unique molecular identifiers (UMIs), $x_{t}$ corresponds directly to the read count associated with cluster *t*. To better understand how data preparation affects DU analysis, we explore two types of modeling. The first type models TSS usage at the single-cell resolution, while the other type models TSS usage at the bulk level by aggregating data from each single-cell sample into a pseudo-bulk sample.

Suppose there are *J* single-cell samples, *K* biological conditions, and $I_{j}$ cells in sample *j*. We consider one TSS cluster each time. For single-cell level modeling, we denote $x_{ij}$ as the read count of a specific TSS cluster in cell $i\;(i=1,2,\ldots ,I_{j})$ from sample *j* ( *j* = 1 , 2 , *…* , *J* ) , and we use $k_{j}\in \{1,2,\ldots ,K\}$ to denote the condition of this sample. Then we assume that $x_{ij}$ follows a binomial distribution,


$$\begin{array}{ccc} x_{ij}∼\text{Binomial}(y_{ij},\theta_{j})\;, & & \end{array}$$ (2)

where $y_{ij}$ is the overall read count of all TSS clusters on the same gene for cell *i* in sample *j*, and $\theta_{j}\in [0,1]$ is the (unobserved) true usage of the TSS cluster of interest in sample *j*. Then, we further assume a linear relationship between the logit of $\theta_{j}$ and sample/condition-level variables:


$$\begin{array}{ccc} \text{logit}(\theta_{j})=\beta_{0}+z_{j}^{⊺}\beta +\phi_{j}\;, & & \end{array}$$ (3)

where $\beta_{0}$ is an intercept, and $z_{j}$ is a vector of the condition variable(s) and other available covariates. In the special case where no other covariates are available, $z_{j}$ is a binary vector of size  ( *K* − 1 ) × 1. Suppose we select condition 1 as the baseline condition (without loss of generality). If $k_{j}=1$, the elements of $z_{j}$ are all zero values; if $k_{j}>1$, the elements of $z_{ij}$ are all zero values except for the $\left(k_{j}\;-\;1\right)$-th element. *β* denotes the corresponding coefficient(s) to be estimated. $\phi_{j}$ is the sample-level random effect for sample *j*, which is used to account for the sample-level heterogeneity. We assume $\phi_{j}∼\text{N}(0,\sigma^{2}).$ The above model can be estimated using the “glmer” function from the R package lme4 [49]. For the DU analysis, we test the significance of usage change based on the statistical significance of *β*, using the “anova” function from the lme4 package.

For pseudo-bulk level modeling, we let


$$\begin{array}{ccc} {\tilde{x}}_{j}=\overset{I_{j}}{\underset{i=1}{\sum }}x_{ij}\; & & \end{array}$$ (4)

be the pseudo-bulk level read count of the TSS cluster of interest, and


$$\begin{array}{ccc} {ỹ}_{j}=\overset{I}{\underset{i=1}{\sum }}y_{ij}\; & & \end{array}$$ (5)

be the pseudo-bulk level read count of the gene. Then, we assume a Binomial model


$$\begin{array}{ccc} {\tilde{x}}_{j}∼\text{Binomial}({ỹ}_{j},\theta_{j})\;. & & \end{array}$$ (6)

where $\theta_{j}$ denotes the true TSS cluster usage of sample *j*. We further assume the same link function as specified in formula (3), indicating a linear relationship between the logit of $\theta_{j}$ and sample/condition-level variables. The pseudo-bulk level model is also estimated and tested by the lme4 package.

### Simulation study of DU analysis

**(1) Generation of simulated data**

In order to generate simulated TSS cluster expression data with ground truth information on TSS cluster usage, we first simulated the read counts of genes, and then simulated the read counts of TSS clusters. We considered the simulation setting of two biological conditions (i.e., cell types), with J(5,10,15,20,or25) single-cell samples in each condition, and I(100or500) single-cells in each sample. To simulate gene-level read counts, we utilized the R package scDesign2 [50], which learns distributional characteristics of single-cell gene expression levels from real data. In this study, we used scDesign2 to learn gene expression parameters from the real single-cell gene expression levels of the healthy donors from the COVID-19 dataset [33]. To account for the potential gene expression changes between biological conditions, which is a common counfouding factor in DU analysis, we focused on two different cell types, B cells and activated T cells. Before generating synthetic gene expression data for these two celltypes, we used the approach below to filter genes. First, we only retained genes expressed in at least 1*%* of cells in all five samples. Second, we selected the top 2,000 genes with the highest detection rates in B cells and activated T cells. Given the filtered real data, we could use scDesign2 to generate synthetic gene expression data for varying sample sizes and cell numbers.

To simulate TSS-cluster-level count data, we assumed there were two TSS clusters on each gene. For the first TSS cluster on a gene, we let $\gamma_{ijk}$ represent its usage in cell *i*  ( *i* = 1 , 2 , *…* , *I* )  from sample *j*  ( *j* = 1 , 2 , *…* , *J* )  under biological condition *k*  ( *k* = 1 , 2 ) . The usage was randomly drawn from a Beta distribution:


$$\begin{array}{ccc} \gamma_{ijk}∼\text{Beta}(\alpha =\alpha_{jk},\beta =5)\;, & & \end{array}$$ (7)

where $\alpha_{jk}$ and *β* were the two shape parameters. Therefore, the corresponding mean TSS usage was $\mu_{jk}=\frac{\alpha_{jk}}{\alpha_{jk}+\beta }\;(\alpha_{jk}=\frac{\beta \mu_{jk}}{1-\mu_{jk}})$. The values of $\mu_{jk}$ and $\alpha_{jk}$ were determined depending on the null and alternative hypotheses. We assumed that TSS clusters on 1,000 genes followed the null hypothesis and TSS clusters on the other 1,000 genes followed the alternative hypothesis.

Under the null hypothesis, we assumed that the expected TSS cluster usage was the same in the two biological groups. Thus, we first generated a baseline usage, *a*, from Uniform ( 0 . 1 , 0 . 9 ) . In addition, to account for sample-level variability, we drew $\tau_{jk}$ for sample *j* in group *k*:


$$\begin{array}{ccc} \tau_{jk}∼(1-\pi )\text{Uniform}(-0.1,0.1)+\frac{\pi }{2}\text{Uniform}(c_{1},c_{2})+\frac{\pi }{2}\text{Uniform}(-c_{2},-c_{1})\;, & & \end{array}$$ (8)

where *π* represented the possibility of a sample being an outlier regarding the usage of this TSS cluster, and $c_{1}$ and $c_{2}$ represented the deviation of outliers from the expected values. We considered *π* ∈ { 0 . 1 , 0 . 2 , 0 . 3 , 0 . 4 }  and $c_{1}=0.1,c_{2}=0.2$ (small outlier deviation) or $c_{1}=0.2,c_{2}=0.3$ (large outlier deviation). The expected usage of the first TSS cluster in sample *j* of group *k* was finally determined as follows:


$$\begin{array}{ccc} \mu_{jk}=\{\begin{array}{cc} (a+\tau_{jk})\;,\; & \text{if }0<(a_{k}+\tau_{jk})<1\;;\\ 1\;,\; & \text{if }(a_{k}+\tau_{jk})\geq 1\;;\\ 0\;,\; & \text{if }(a_{k}+\tau_{jk})\leq 0\;. \end{array} & & \end{array}$$ (9)

Under the alternative hypothesis, we assumed that the expected TSS cluster usage was different in the two biological groups. We generated $a_{1}∼\text{Uniform}(0.1,0.4)$ as the baseline usage in group 1, and $a_{2}=a_{1}\;+\;d$ as the baseline usage in group 2, where *d* ∼ Uniform ( 0 . 1 , 0 . 5 ) . As for the sample-level effect, we assume $\tau_{jk}$ follows a mixture distribution:


$$\begin{array}{ccc} \tau_{j,k=1} & ∼(1-\pi )\text{Uniform}(-0.1,0.1)+\pi \text{Uniform}(c_{1},c_{2}); & \end{array}$$ (10)

$$\begin{array}{ccc} \tau_{j,k=2} & ∼(1-\pi )\text{Uniform}(-0.1,0.1)+\pi \text{Uniform}(c_{1},c_{2})\;, & \end{array}$$ (11)

where $\pi ,c_{1},\text{and}\;c_{2}$ took the same values as under the null hypothesis. Then, the expected usage of the first TSS cluster in sample *j* of group *k* was determined as follows:


$$\begin{array}{ccc} \mu_{jk}=\{\begin{array}{cc} (a_{k}+\tau_{jk})\;,\; & \text{if }0<(a_{k}+\tau_{jk})<1\;;\\ 1\;,\; & \text{if }(a_{k}+\tau_{jk})\geq 1\;;\\ 0\;,\; & \text{if }(a_{k}+\tau_{jk})\leq 0\;. \end{array} & & \end{array}$$ (12)

Once $\mu_{jk}$’s were obtained, we draw TSS usage ($\gamma_{ijk}$) for individual cells based on formula (7). The usage of the other TSS cluster on the same gene was automatically obtained as $1-\gamma_{ijk}$. Given the single-cell level TSS usage, the TSS-cluster-level read counts were generated by a Binomial distribution with the gene-level read count as the sample size.

**(2) Alternative methods for DU analysis**

Other than the two Binomial GLMM models included in scTSS toolkit, we applied five alternative methods for DU analysis of the simulated data, including DEXSeq [37], IDEAS [39], MAST [38], and the Wilcoxon rank sum test (both at single-cell and pseudo-bulk level). For all methods, we applied the Benjamini-Hochberg procedure to adjust *P*-values for multiple testing.

DEXSeq was initially designed for detecting alternative transcription usage for bulk RNA-seq data. Thus, we formed a pseudo-bulk sample for each single-cell sample in the simulated data. When applying DEXSeq for DU analysis, we followed its tutorial and set the formula as “sample + TSS + condition:TSS” for the full model and “sample + TSS” for the reduced model (null hypothesis).

IDEAS was originally designed for differential gene expression analysis based on multiple single-cell samples. It first estimates the distribution of gene expression in each individual, and then performs the test by comparing within-group distances of individuals with between-group distances. However, IDEAS cannot be directly applied to TSS usage data, so we made the following modifications for DU analysis. First, we converted the TSS cluster counts into TSS cluster usage. Second, we used the Wasserstein-1 distance to measure the difference between the empirical CDFs of TSS cluster usage between any two single-cell samples. Third, we performed the permutation 2000 times for each TSS cluster to obtain its *P*-value.

MAST was designed for differential gene expression analysis based on single-cell RNA-seq data. When applying MAST on TSS cluster counts for DU analysis, in the “zlm” function, we set the formula for the full model as “sample+condition”. When conducting the likelihood ratio test to obtain the *P*-values, we set “doLRT=condition”.

To perform the Wilcoxon rank sum test at the pseudo-bulk level, we treated the cells from each biological group and single-cell sample as a pseudo-bulk sample, and added up the read counts of a TSS cluster across the cells before calculating TSS usage. To perform Wilcoxon rank sum test at the single-cell level, we calculated the TSS cluster usage in individual cells. The Wilcoxon rank sum test was directly applied to the calculated TSS cluster usage.

### Real data analysis

The raw sequences in the COVID-19 dataset were processed by Cell Ranger software (“count” mode) to align reads to the reference genome using default parameters and obtain BAM files. The GRCh38 reference genome was downloaded from 10x Genomics’ website (References-2020-A, https://cf.10xgenomics.com/supp/cell-exp/refdata-gex-GRCh38-2020-A.tar.gz). Four methods, including CAGEr [17], TSSr [18], TSRexploreR [19], and SCAFE [26], were applied via the scTSS pipeline to perform TSS cluster prediction and DU analysis. For CAGEr, the “method” parameter was set as “distclu” in the “clusterCTSS” function, and other parameters were set to default. For TSSr, we first ran the “filterTSS” function followed by the “clusterTSS” function with default parameters. For TSRexploreR, we set “threshold=3” and “max_distance=25” in the “tss_clustering” function, which were used in the software tutorial. For SCAFE, we followed the standard workflow in its tutorial, and used the default pretrained logistic regression model in the “tool.cm.filter” function. For the motif analysis on differentially used TSSs between activated T cells and B cells, we used the motif discovery algorithm (findMotif.pl) from Homer [51]. For each top differentially used TSS cluster, we used the 150 bp upstream of the dominant TSS as the region for motif identification. The background region was set as the 150 bp upstream of the dominant TSS in all the predicted TSS clusters used in the DU analysis. The “len” parameter was set to 8. To analyze the subpopulations of naïve T cells in the five healthy samples by TSS usage, we performed TSS prediction on each sample and TSS merging across samples. Then, we converted the TSS counts to TSS usage for each TSS cluster. Lastly, we followed the clustering procedure in Seurat [52] to identify cell subpopulations based on TSS usage.

For the Arthritis dataset, the raw sequences were aligned using the same approach as described for the COVID-19 dataset. CAGEr, TSSr and TSRexploreR were applied via the scTSS pipeline to perform TSS cluster prediction and DU analysis. The parameter settings were the same as described above. SCAFE was excluded as it is not applicable to near-site data.

In DU analysis on both datasets, we applied the Benjamini-Hochberg procedure to adjust *P*-values for multiple testing.

### Calculation of precision, recall, and F1 score

The gene-level precision was defined for each gene based on the proportion of predicted TSS clusters that were observed in the annotation. We considered a predicted TSS cluster to be observed if it overlapped with an annotated TSS on the same gene. The gene-level recall was defined for each gene based on the proportion of annotated TSSs that were predicted by scTSS. We considered an annotated TSS as being predicted if it was included by a predicted TSS cluster on the same gene. Lastly, the overall precision (*P*) and recall (*R*) for a single-cell sample were obtained by calculating the average of gene-level precision and recall, respectively. In addition, the F1 score for a single-cell sample was defined as


$$\begin{array}{ccc} F1=2\times \frac{P\times R}{P+R}\;. & & \end{array}$$ (13)

## Supporting information

S1 Table Feature comparison between bioinformatic tools for TSS clustering. Mapped TSS: the genomic location of a TSS identified by directly mapping the 5’ end of Read 1 to the genome; mapped TSS table: a table obtained by tabulating the frequency of all mapped TSSs.
(PDF)

S1 Fig TSS prediction results of the COVID-19 dataset. (**A**) Accuracy of TSS cluster prediction based on one-annotated-TSS genes in the FANTOM5 annotation. The precision, recall, and F1 scores were first calculated for each sample, and then averaged across samples. The error bars indicate the standard deviation of the scores. (**B**) The nucleotide proportion at -1 and +1 positions of predicted dominant TSSs for each of the TSS cluster prediction method. (**C**) Gene numbers with varying number of predicted TSS clusters based on the prediction of SCAFE. The bars represent mean value across samples, and the error bars represent the the standard deviation. (**D**) The distribution of TSS cluster width in each sample based on the prediction from SCAFE. (**E**) The relative abundance of different types of predicted dominant TSSs based on SCAFE. The abundance of the “intergenic” category was very low, making it visually inconspicuous in the figure.
(TIF)

S2 Fig TSS prediction results based on Read 2 from the COVID-19 dataset. (**A**) The distribution of genomic distance between predicted TSS cluster centers and the corresponding annotated TSSs using genes with one annotated TSS. A positive distance means the predicted dominant TSS is on the 5’ side of the annotated TSS and vice versa. (**B**) Accuracy of TSS cluster prediction based on all genes in the FANTOM5 annotation. The precision, recall, and F1 scores were first calculated for each sample, and then averaged across samples. The error bars indicate the standard deviation of the scores. (**C**) The accuracy of TSS clusters predicted on Read 2 by TSSr. TSS clusters identified based on Read 1 by TSSr were treated as the reference to calculate these scores. (**D**) The proportion of different types of predicted TSS cluster centers based on TSSr. The classification of center locations was based on the GRCh38 reference. When the original near-site clusters were located in an intronic region, indicating missing annotations, we could not confirm the locations of genuine TSS centers. Therefore, we excluded those clusters from the categorization.
(TIF)

S3 Fig TSS prediction results on the near-site Arthritis dataset. (**A**) Accuracy of TSS cluster prediction based on one-annotated-TSS genes in the FANTOM5 annotation. The precision, recall, and F1 scores were first calculated for each sample, and then averaged across samples. The error bars indicate the standard deviation of the scores. (**B**) The distribution of TSS cluster width in each sample based on the prediction of TSSr. (**C**) The distribution of the distance between the 5’ end of Read 2 and the annotated TSS for genes with only one annotated TSS (based on FANTOM5). (**D**) The relative abundance of different types of predicted TSS clusters based on TSSr. The abundance of the “intergenic” category was very low, making it visually inconspicuous in the figure.
(TIF)

S4 Fig Simulation study of DU analysis with 100 cells per sample. (**A**) Comparison of type I error rates between seven TSS DU testing methods given various sample size, outlier frequency (*π*), and degree of outlier deviation. (**B**) Comparison of statistical power between seven TSS DU testing methods. The results of bulk-level and cell-level Binomial GLMMs were virtually identical, resulting in overlapping lines.
(TIF)

S5 Fig Running time of the seven TSS DU testing methods given various sample sizes and cell numbers per sample. (TIF)

S6 Fig Motif analysis of promoter sequences based on top differentially used TSSs in the COVID-19 dataset. The top 10 enriched known motifs for ACT and B cells are displayed. The color of the dots represents the adjusted *P*-value, and the size of the dots represents the percentage of target sequences detected with known motifs.
(TIF)

S7 Fig Comparison between DU analysis and DGE analysis. (**A**) Heatmap of normalized gene expression in ACT and B cells from the COVID-19 dataset. For each TSS cluster in Fig 5A, we visualized the normalized expression levels of the corresponding gene in this heatmap. (**B**) A contingency table was constructed to compare genes with TSS clusters showing differential usage against genes identified as differentially expressed between ACT and B cells (COVID-19 dataset). (**C**) Heatmap of normalized gene expression in SF and PB samples from the Arthritis dataset. For each TSS cluster in Fig S9, we visualized the normalized expression levels of the corresponding gene in this heatmap. (**D**) A contingency table for genes with TSS clusters showing differential usage against genes identified as differentially expressed between SF and PB samples (Arthritis dataset).
(TIF)

S8 Fig Comparison between DEXSeq and Binomial GLMM on the COVID-19 dataset. (**A**) The distribution of *P*-values obtained by DEXSeq and Binomial GLMM for the DU test between activated T cells and B cells. (**B**) The Venn diagram of identified differential TSS clusters by Binomial GLMM and DEXSeq. A TSS cluster is differential if the BH corrected *P*-value is below 0.05. (**C**) The ranking of TSS clusters yielded by Binomial GLMM and DEXSeq. The TSS clusters are ranked based on their *P*-values.
(TIF)

S9 Fig Differential TSS usage analysis between T cells from PB and SF samples in arthritis patients. (**A**) Heatmap of normalized average TSS usage in arthritis patients. For each TSS cluster, its sample-specific usage was first calculated by taking the average across all cells in that sample. Then, the average usage was normalized across samples using the min-max normalization. (**B**) Cumulative distribution function (CDF) of TSS usage of genes *CHCHD5* and *UBR2* under two conditions (PB and SF). Each line represents the corresponding TSS usage of one patient. TSS cluster chr2:*CHCHD5*:112,585,869-112,585,937:+ had higher expression in PB samples, while chr6:*UBR2*:42,563,957-42,564,104:+ had higher expression in SF samples.
(TIF)

S10 Fig Comparison between DEXSeq and Binomial GLMM on the Arthritis data. (**A**) The distribution of *P*-values obtained by DEXSeq and Binomial GLMM for the DU test betweenT cells in PB and SF samples. (**B**) The Venn diagram of identified differential TSS clusters by Binomial GLMM Binomial and DEXSeq. A TSS cluster is differential if the BH corrected *P*-value is below 0.05. (**C**) The ranking of TSS clusters yielded by Binomial GLMM and DEXSeq. The TSS clusters are ranked based on their *P*-values.
(TIF)

S11 Fig Heatmap of uniquely identified differential TSS clusters by Binomial GLMM and DEXseq from the Arthritis data. (**A**) Relative usage of differential TSS clusters uniquely identified by Binomial GLMM. For each TSS cluster, its sample-specific usage was first calculated by taking the average across all cells in that sample. Then, the average usage was normalized across samples using the min-max normalization. (**B**) Relative usage of differential TSS clusters uniquely identified by DEXSeq.
(TIF)

S12 Fig Cumulative distribution function (CDF) of TSS usage of genes in the Arthritis data. (**A**) *MAZ*. (**B**) *PHACTR4*. Each row of CDF curve represents the TSS usage distribution of one patient. If a gene was not detected in a patient, the corresponding panel would be empty. Both TSS clusters had higher usage in the PB samples.
(TIF)

S13 Fig Cumulative distribution function (CDF) of TSS usage of genes in the Arthritis data. (**A**) *IGKC*. (**B**) *ERI1*. Each row of CDF curve represents the TSS usage distribution of one patient. If a gene was not detected in a patient, the corresponding panel would be empty. Both TSS clusters had higher usage in the PB samples.
(TIF)

S14 Fig Sub-sampling analysis of DU tests. (**A**) Cell sub-sampling analysis of the COVID-19 dataset. The bars indicate the average number of differentially used TSS clusters identified by different methods across the 10 repeats. The error bars indicate the standard deviation of the numbers. (**B**) Sample sub-sampling analysis of the COVID-19 dataset. (**C**) Cell sub-sampling analysis of the Arthritis dataset. (**D**) Sample sub-sampling analysis of the Arthritis dataset.
(TIF)
