# Supplementary material for: Predicting and comparing transcription start sites in single cell populations
Source: PLoS Comput Biol. 2025 Apr 3;21(4):e1012878. doi: 10.1371/journal.pcbi.1012878 (PMC11968111; doi:10.1371/journal.pcbi.1012878)
Supplement: S1 Table — Mapped TSS: the genomic location of a TSS identified by directly mapping the 5’ end of Read 1 to the genome; mapped TSS table: a table obtained by tabulating the frequency of all mapped TSSs. (PDF) [file pcbi.1012878.s015.pdf]

S1 Table for "Predicting and comparing transcription start sites in single cell populations"

Table A: Feature comparison between bioinformatic tools for TSS clustering. Mapped TSS: the genomic location of a TSS identified by directly mapping the 5' end of Read 1 to the genome; mapped TSS table: a table obtained by tabulating the frequency of all mapped TSSs.

**Method**	**Input data resolution**	**Input data type**	**TSS clustering method**	**Filtering criteria for TSS clusters**	**Differential TSS cluster usage analysis**
CAGEr	Bulk	BAM or mapped TSS table	1) Distance-based clustering with fixed threshold between neighboring TSSs; 2) paraclu [1]	Length and expression levels of TSS clusters	N/A
TSRexploreR	Bulk	BAM or mapped TSS table	Distance-based clustering with fixed threshold between neighboring TSSs	Length and expression levels of TSS clusters	DEseq2 [2]
TSSr	Bulk	BAM or mapped TSS table	Sliding window approach with fixed window size	Number of supporting reads; signal strength and distance of TSS clusters	For each gene, the two most highly expressed TSS clusters were selected. A promoter shift score is calculated for the two selected TSS clusters and the chi-square test is implemented to assess statistical significance [3].
SCAFE	Single-cell	BAM	paraclu [1]	Classification based on a logistic regression model trained on ATAC-seq data paired with 5' single-cell RNA-seq.	N/A
CamoTSS[1]	Single-cell	BAM	Hierachical clustering based on the average linkage	1) Classification based on a logistic regression model (similar to SCAFE); 2) Classification based on a convolutional neural network trained on ATAC-seq data paired with 5' single-cell RNA-seq.	BRIE2 [4]

[1]The CamoTSS method [5] was also specifically designed for 5' scRNA-seq on-site data. We did not include CamoTSS in our numerical comparison due to an error message encountered in the TSS clustering step.

References

[1] Martin C Frith, Eivind Valen, Anders Krogh, Yoshihide Hayashizaki, Piero Carninci, and Albin Sandelin. A code for transcription initiation in mammalian genomes. *Genome research*, 18(1):1–12, 2008.

[2] Michael I. Love, Wolfgang Huber, and Simon Anders. Moderated estimation of fold change and dispersion for rna-seq data with deseq2. *Genome Biology*, 15:550, 2014.

[3] Zhaolian Lu and Zhenguo Lin. Pervasive and dynamic transcription initiation in saccharomyces cerevisiae. *Genome Research*, 29(7):1198–1210, 2019.

[4] Yuanhua Huang and Guido Sanguinetti. BRIE2: computational identification of splicing phenotypes from single-cell transcriptomic experiments. *Genome biology*, 22(1):251, 2021.

[5] Ruiyan Hou, Chung-Chau Hon, and Yuanhua Huang. Camotss: analysis of alternative transcription start sites for cellular phenotypes and regulatory patterns from 5'scrna-seq data. *bioRxiv*, pages 2023–04, 2023.
