# Supplementary figures and images for: Predicting and comparing transcription start sites in single cell populations
Source: PLoS Comput Biol. 2025 Apr 3;21(4):e1012878. doi: 10.1371/journal.pcbi.1012878 (PMC11968111; doi:10.1371/journal.pcbi.1012878)

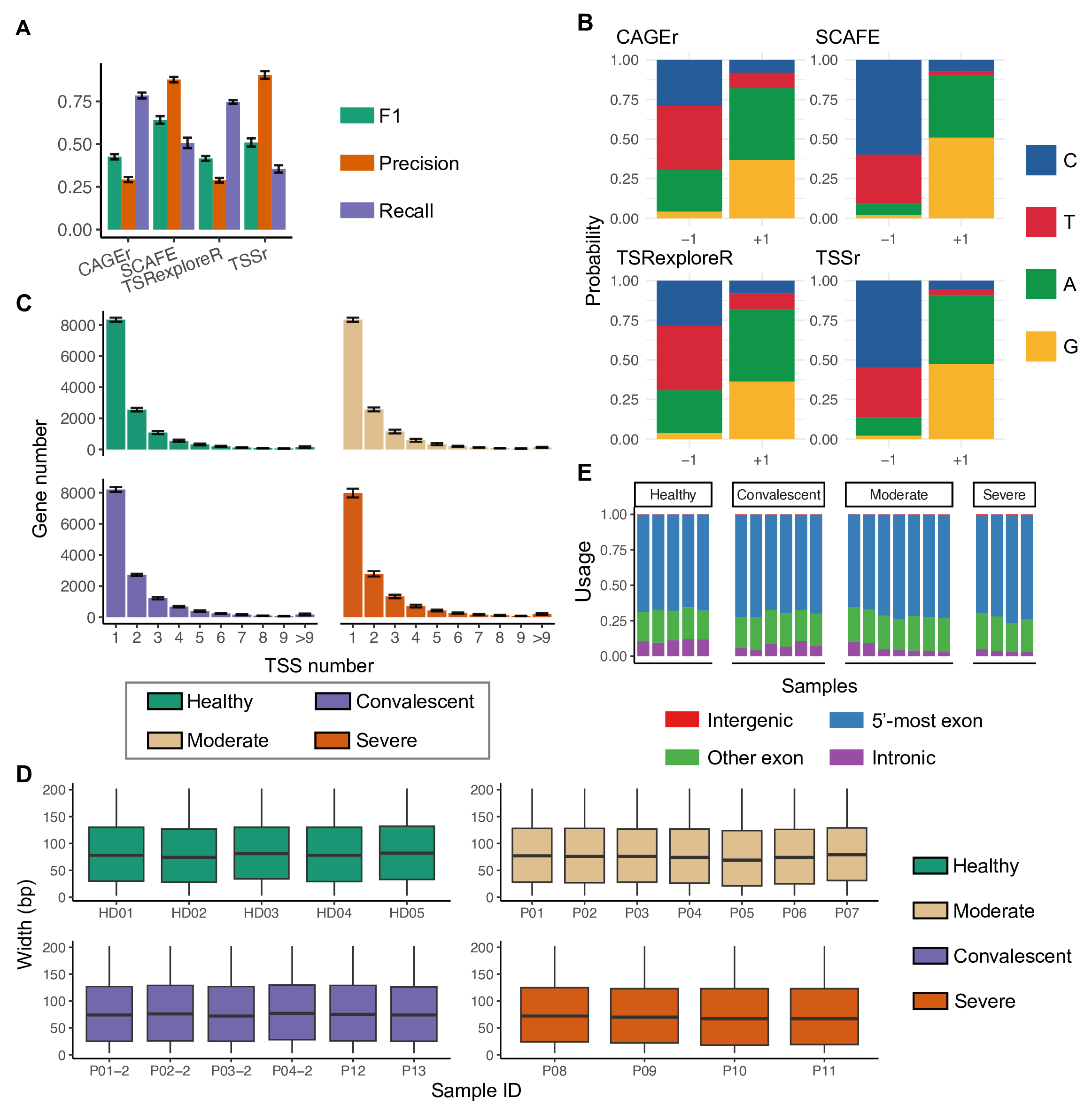

Supplement: S1 Fig — (A) Accuracy of TSS cluster prediction based on one-annotated-TSS genes in the FANTOM5 annotation. The precision, recall, and F1 scores were first calculated for each sample, and then averaged across samples. The error bars indicate the standard deviation of the scores. (B) The nucleotide proportion at -1 and +1 positions of predicted dominant TSSs for each of the TSS cluster prediction method. (C) Gene numbers with varying number of predicted TSS clusters based on the prediction of SCAFE. The bars represent mean value across samples, and the error bars represent the the standard deviation. (D) The distribution of TSS cluster width in each sample based on the prediction from SCAFE. (E) The relative abundance of different types of predicted dominant TSSs based on SCAFE. The abundance of the “intergenic” category was very low, making it visually inconspicuous in the figure. (TIF) [file pcbi.1012878.s001.tif]

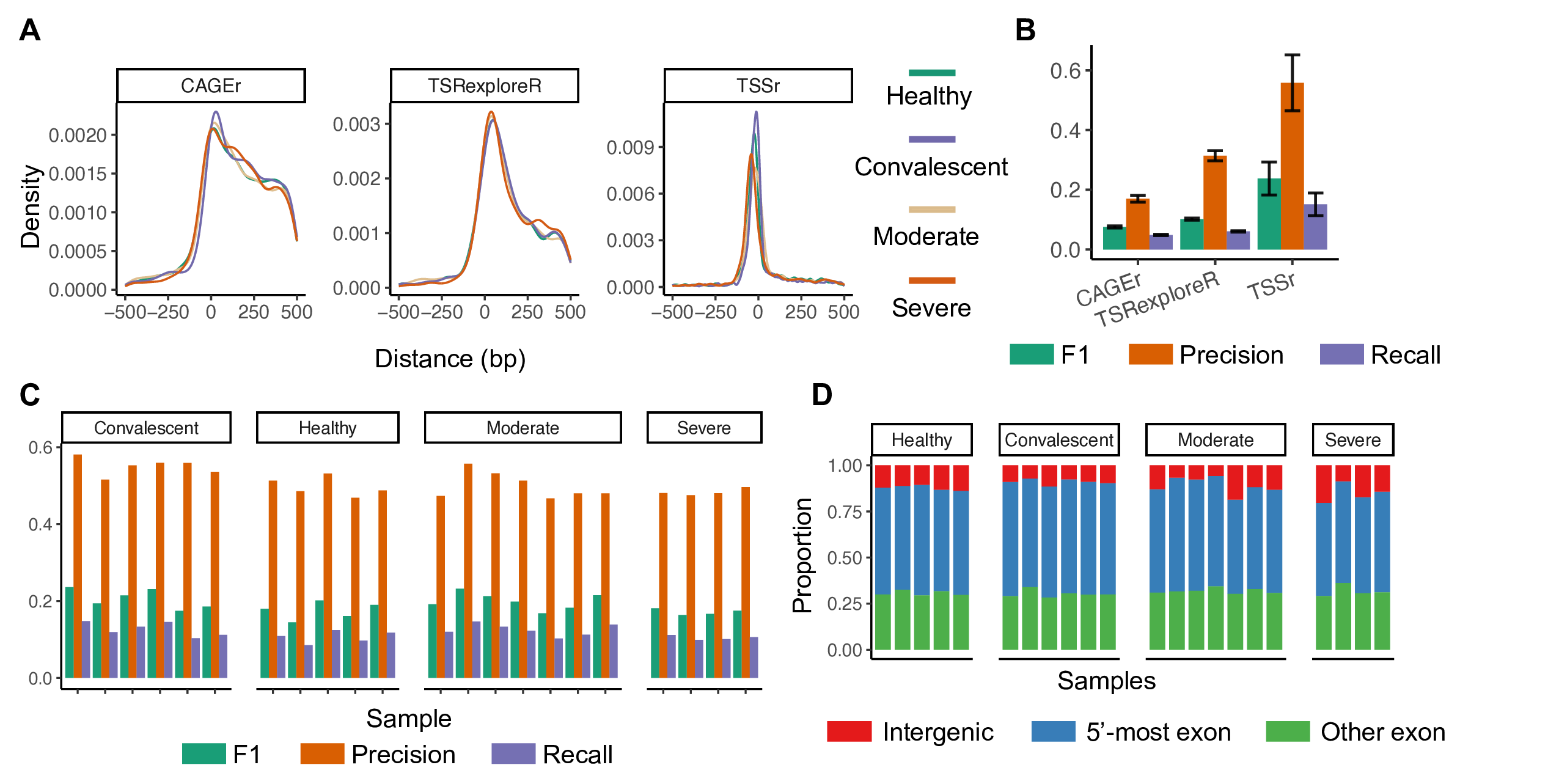

Supplement: S2 Fig — (A) The distribution of genomic distance between predicted TSS cluster centers and the corresponding annotated TSSs using genes with one annotated TSS. A positive distance means the predicted dominant TSS is on the 5’ side of the annotated TSS and vice versa. (B) Accuracy of TSS cluster prediction based on all genes in the FANTOM5 annotation. The precision, recall, and F1 scores were first calculated for each sample, and then averaged across samples. The error bars indicate the standard deviation of the scores. (C) The accuracy of TSS clusters predicted on Read 2 by TSSr. TSS clusters identified based on Read 1 by TSSr were treated as the reference to calculate these scores. (D) The proportion of different types of predicted TSS cluster centers based on TSSr. The classification of center locations was based on the GRCh38 reference. When the original near-site clusters were located in an intronic region, indicating missing annotations, we could not confirm the locations of genuine TSS centers. Therefore, we excluded those clusters from the categorization. (TIF) [file pcbi.1012878.s002.tif]

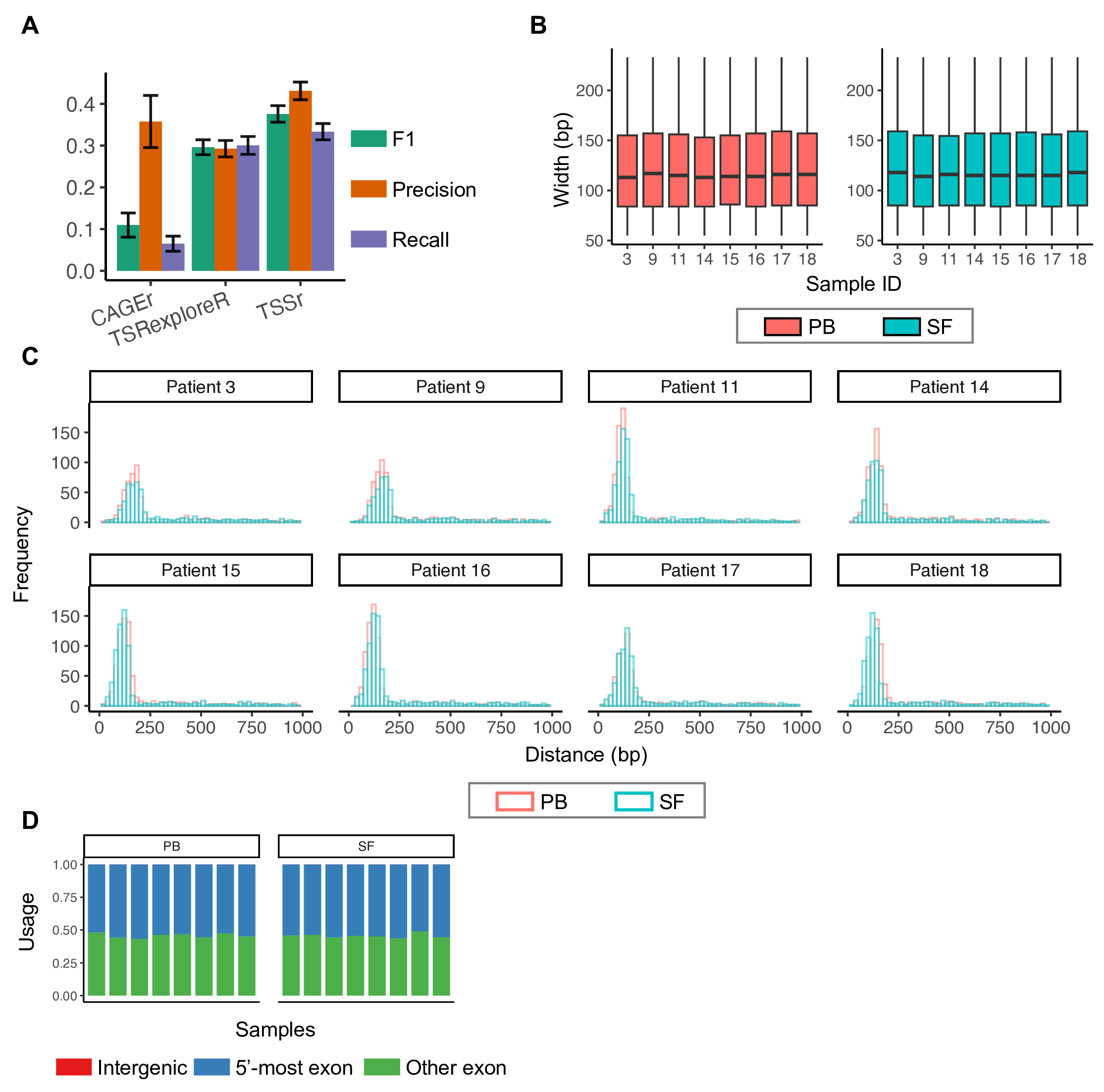

Supplement: S3 Fig — (A) Accuracy of TSS cluster prediction based on one-annotated-TSS genes in the FANTOM5 annotation. The precision, recall, and F1 scores were first calculated for each sample, and then averaged across samples. The error bars indicate the standard deviation of the scores. (B) The distribution of TSS cluster width in each sample based on the prediction of TSSr. (C) The distribution of the distance between the 5’ end of Read 2 and the annotated TSS for genes with only one annotated TSS (based on FANTOM5). (D) The relative abundance of different types of predicted TSS clusters based on TSSr. The abundance of the “intergenic” category was very low, making it visually inconspicuous in the figure. (TIF) [file pcbi.1012878.s003.tif]

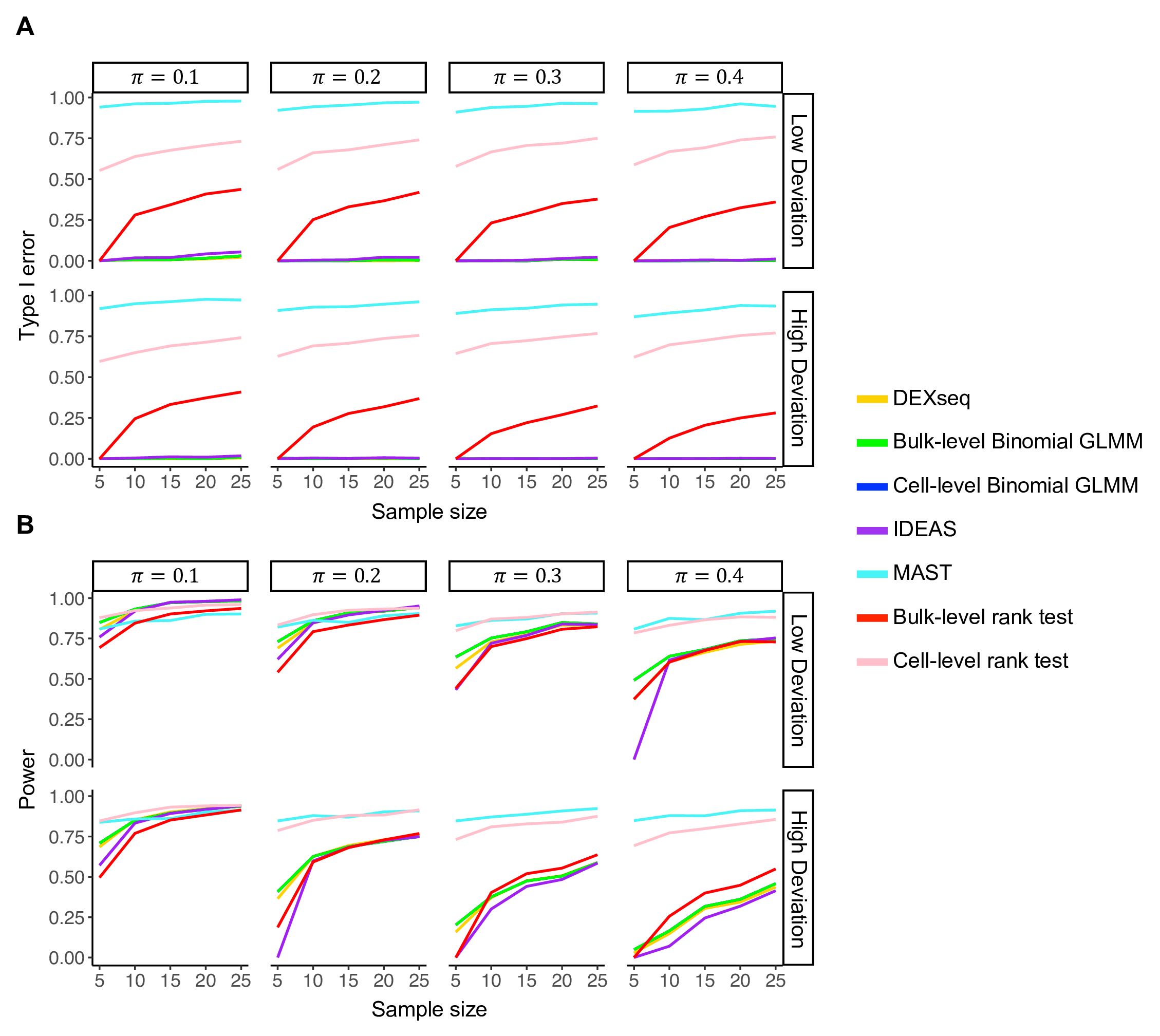

Supplement: S4 Fig — (A) Comparison of type I error rates between seven TSS DU testing methods given various sample size, outlier frequency (π), and degree of outlier deviation. (B) Comparison of statistical power between seven TSS DU testing methods. The results of bulk-level and cell-level Binomial GLMMs were virtually identical, resulting in overlapping lines. (TIF) [file pcbi.1012878.s004.tif]

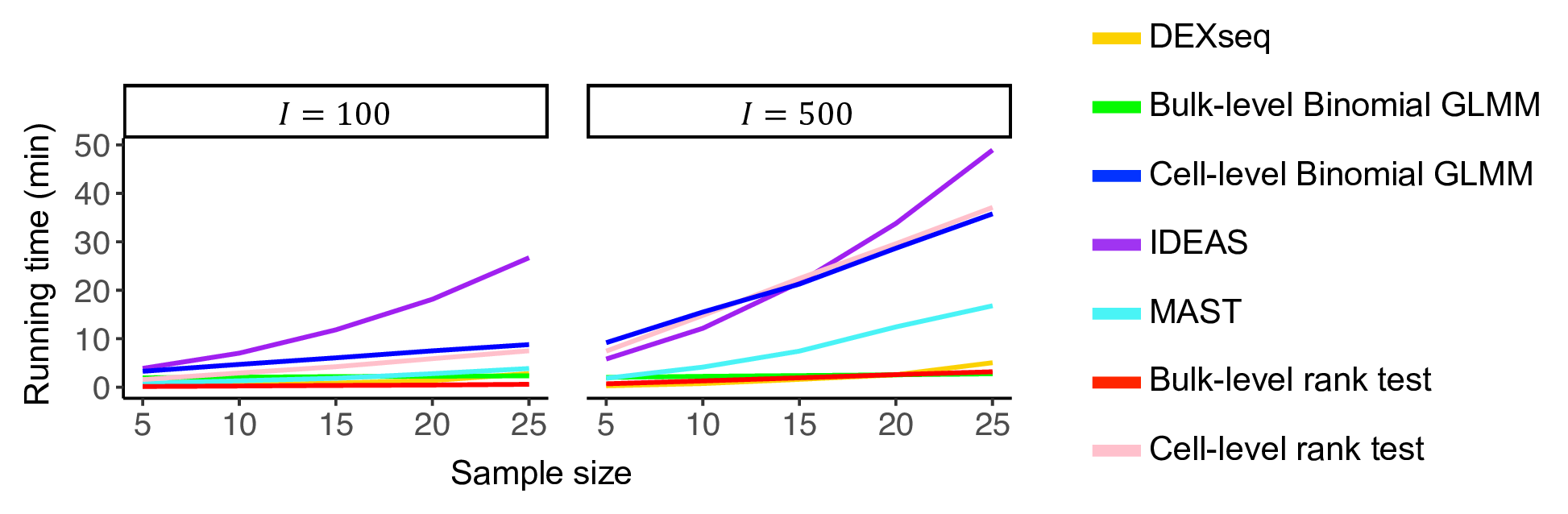

Supplement: S5 Fig — (TIF) [file pcbi.1012878.s005.tif]

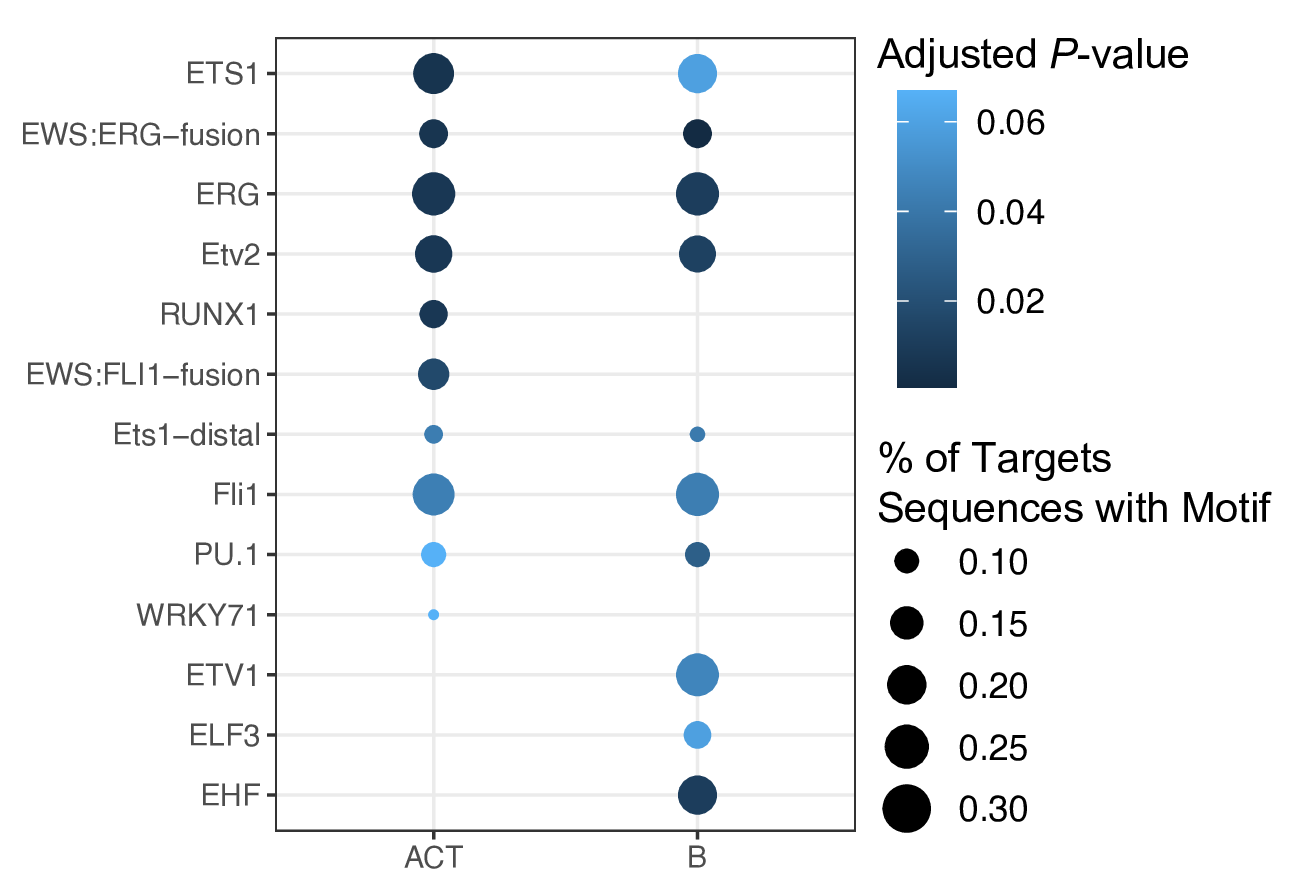

Supplement: S6 Fig — The top 10 enriched known motifs for ACT and B cells are displayed. The color of the dots represents the adjusted P-value, and the size of the dots represents the percentage of target sequences detected with known motifs. (TIF) [file pcbi.1012878.s006.tif]

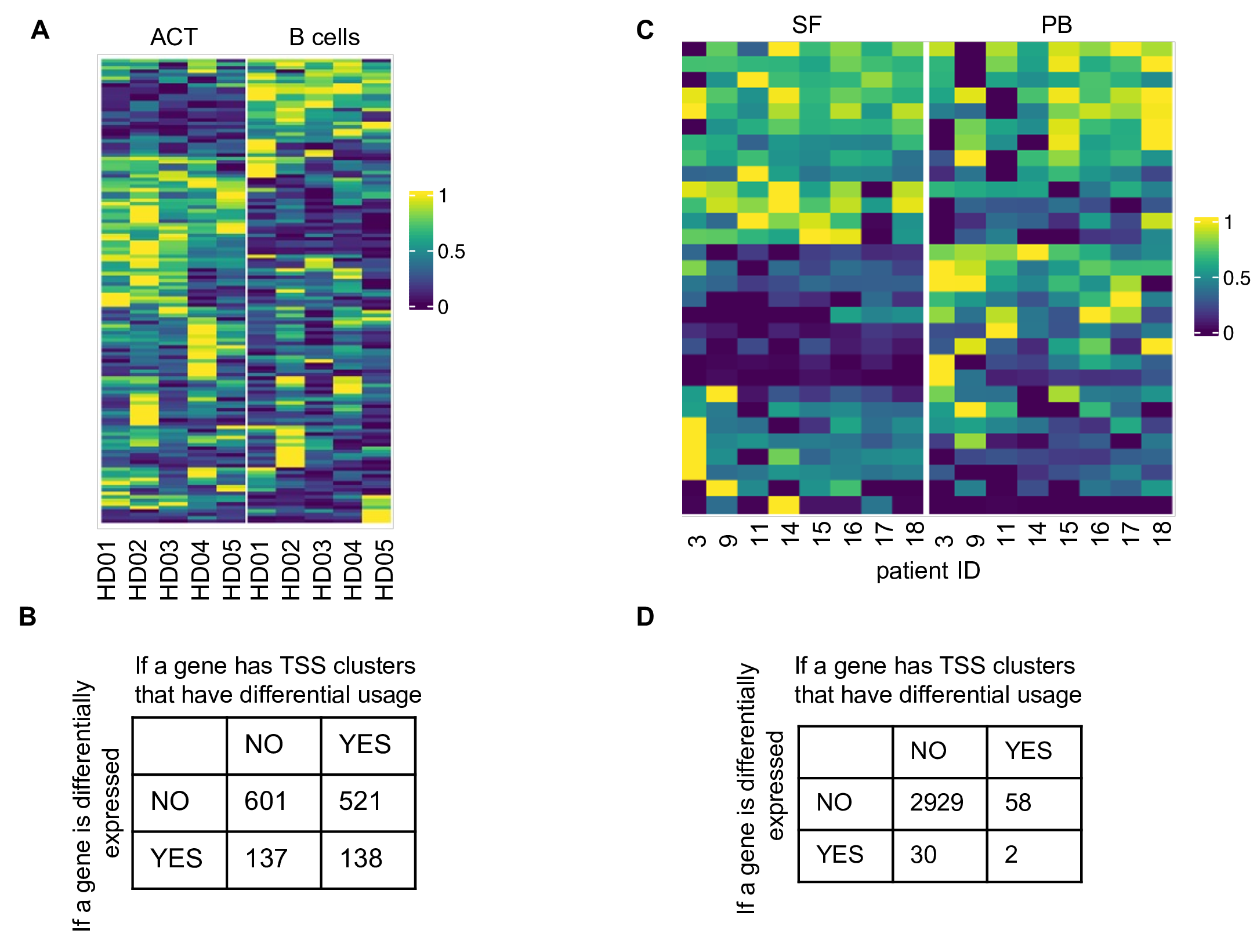

Supplement: S7 Fig — (A) Heatmap of normalized gene expression in ACT and B cells from the COVID-19 dataset. For each TSS cluster in Fig 5A, we visualized the normalized expression levels of the corresponding gene in this heatmap. (B) A contingency table was constructed to compare genes with TSS clusters showing differential usage against genes identified as differentially expressed between ACT and B cells (COVID-19 dataset). (C) Heatmap of normalized gene expression in SF and PB samples from the Arthritis dataset. For each TSS cluster in Fig S9, we visualized the normalized expression levels of the corresponding gene in this heatmap. (D) A contingency table for genes with TSS clusters showing differential usage against genes identified as differentially expressed between SF and PB samples (Arthritis dataset). (TIF) [file pcbi.1012878.s007.tif]

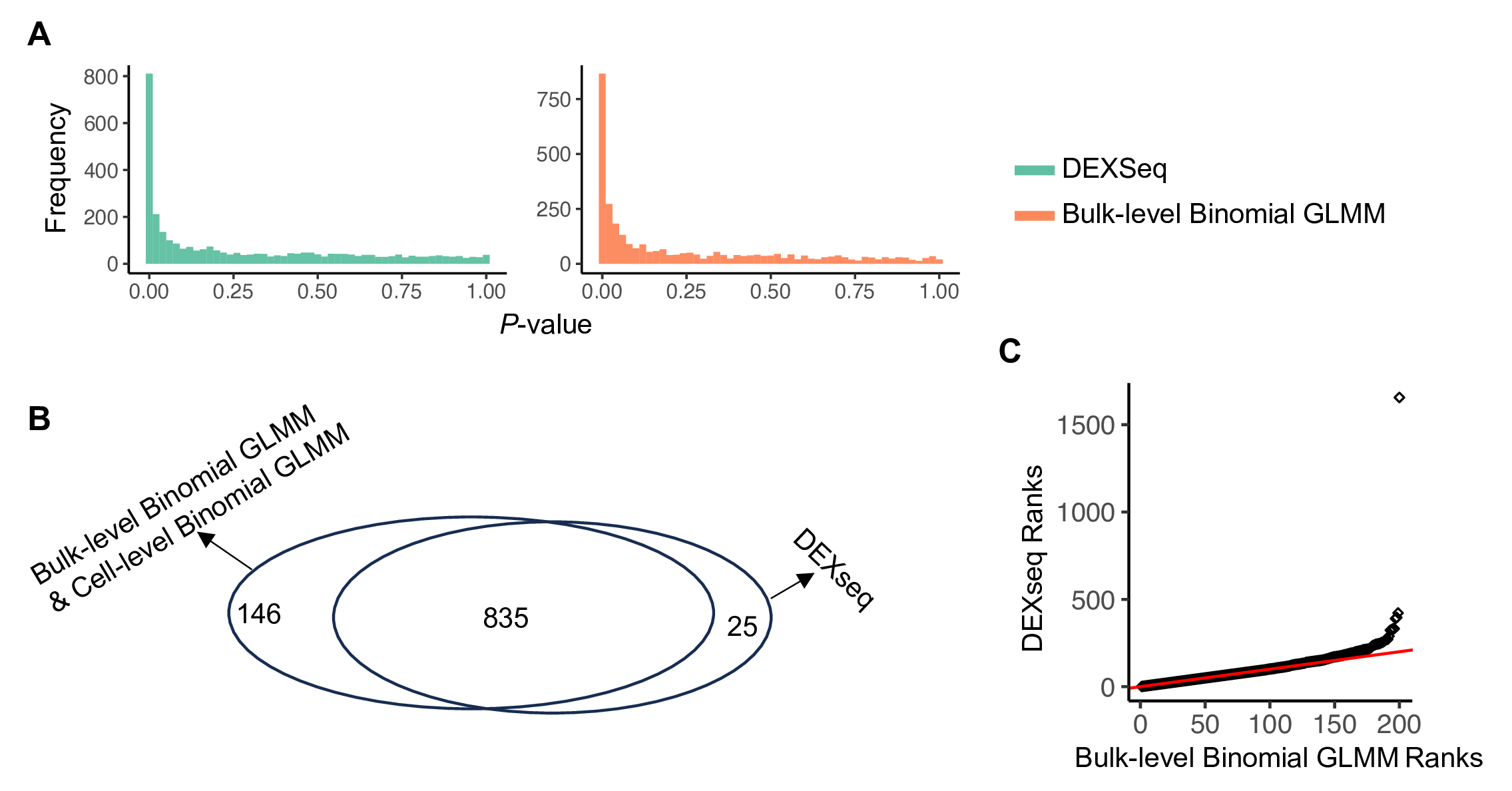

Supplement: S8 Fig — (A) The distribution of P-values obtained by DEXSeq and Binomial GLMM for the DU test between activated T cells and B cells. (B) The Venn diagram of identified differential TSS clusters by Binomial GLMM and DEXSeq. A TSS cluster is differential if the BH corrected P-value is below 0.05. (C) The ranking of TSS clusters yielded by Binomial GLMM and DEXSeq. The TSS clusters are ranked based on their P-values. (TIF) [file pcbi.1012878.s008.tif]

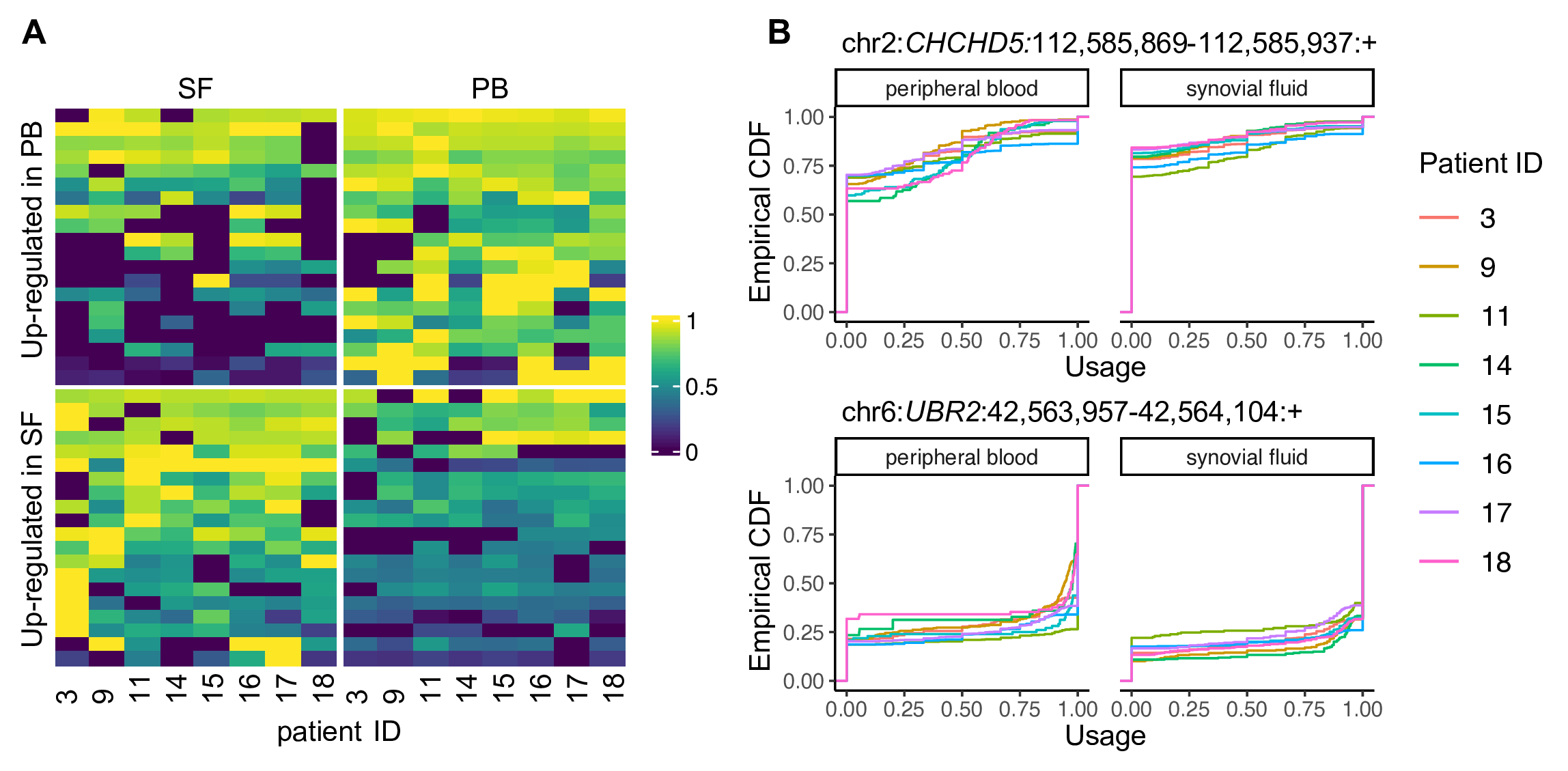

Supplement: S9 Fig — (A) Heatmap of normalized average TSS usage in arthritis patients. For each TSS cluster, its sample-specific usage was first calculated by taking the average across all cells in that sample. Then, the average usage was normalized across samples using the min-max normalization. (B) Cumulative distribution function (CDF) of TSS usage of genes CHCHD5 and UBR2 under two conditions (PB and SF). Each line represents the corresponding TSS usage of one patient. TSS cluster chr2:CHCHD5:112,585,869-112,585,937:+ had higher expression in PB samples, while chr6:UBR2:42,563,957-42,564,104:+ had higher expression in SF samples. (TIF) [file pcbi.1012878.s009.tif]

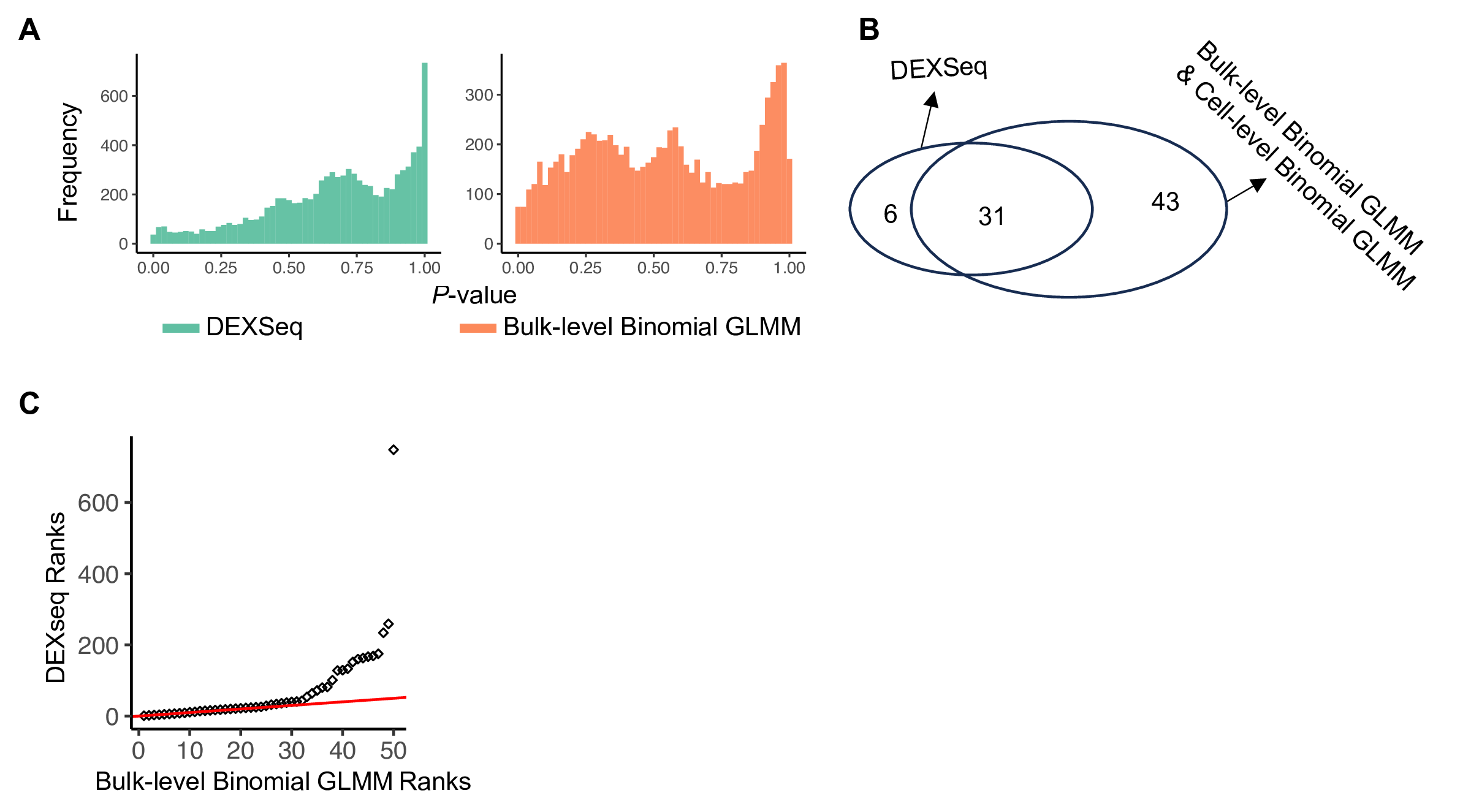

Supplement: S10 Fig — (A) The distribution of P-values obtained by DEXSeq and Binomial GLMM for the DU test betweenT cells in PB and SF samples. (B) The Venn diagram of identified differential TSS clusters by Binomial GLMM Binomial and DEXSeq. A TSS cluster is differential if the BH corrected P-value is below 0.05. (C) The ranking of TSS clusters yielded by Binomial GLMM and DEXSeq. The TSS clusters are ranked based on their P-values. (TIF) [file pcbi.1012878.s010.tif]

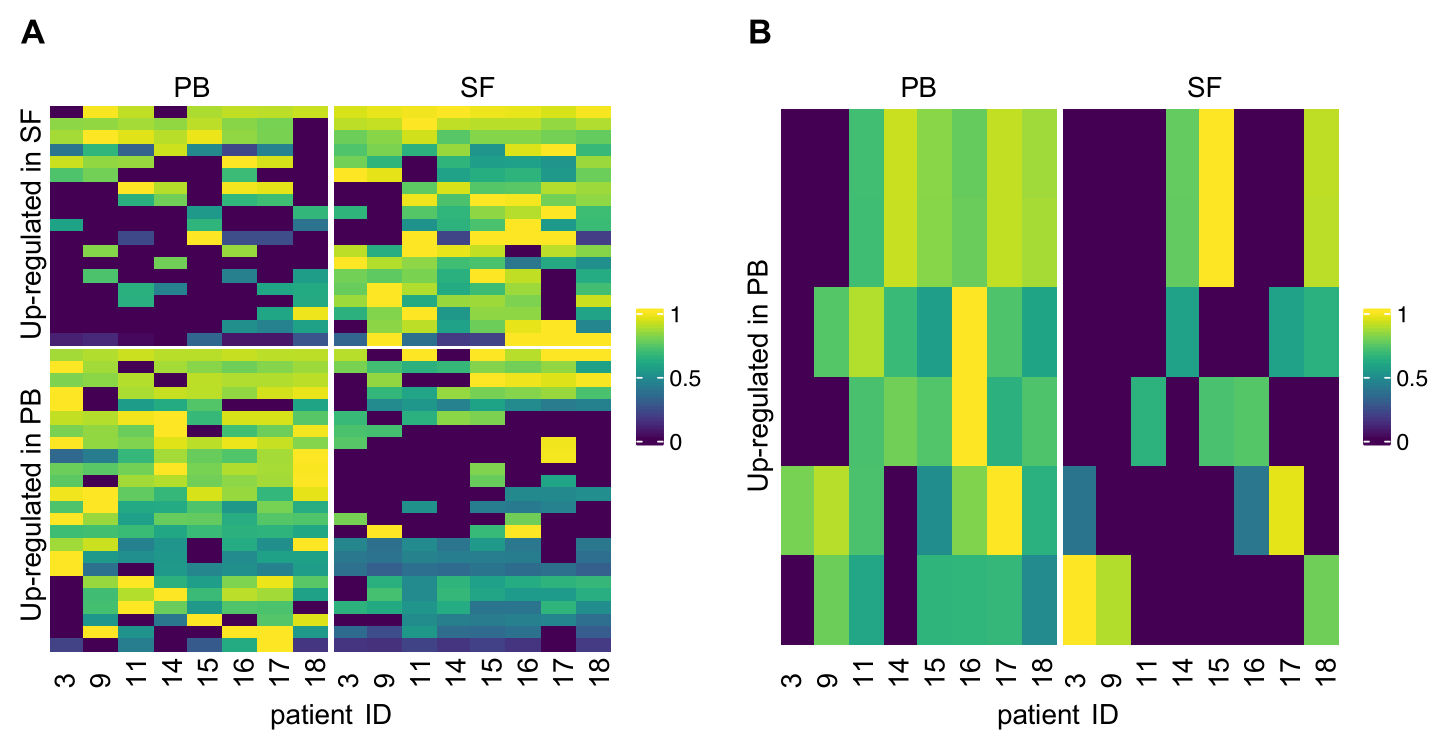

Supplement: S11 Fig — (A) Relative usage of differential TSS clusters uniquely identified by Binomial GLMM. For each TSS cluster, its sample-specific usage was first calculated by taking the average across all cells in that sample. Then, the average usage was normalized across samples using the min-max normalization. (B) Relative usage of differential TSS clusters uniquely identified by DEXSeq. (TIF) [file pcbi.1012878.s011.tif]

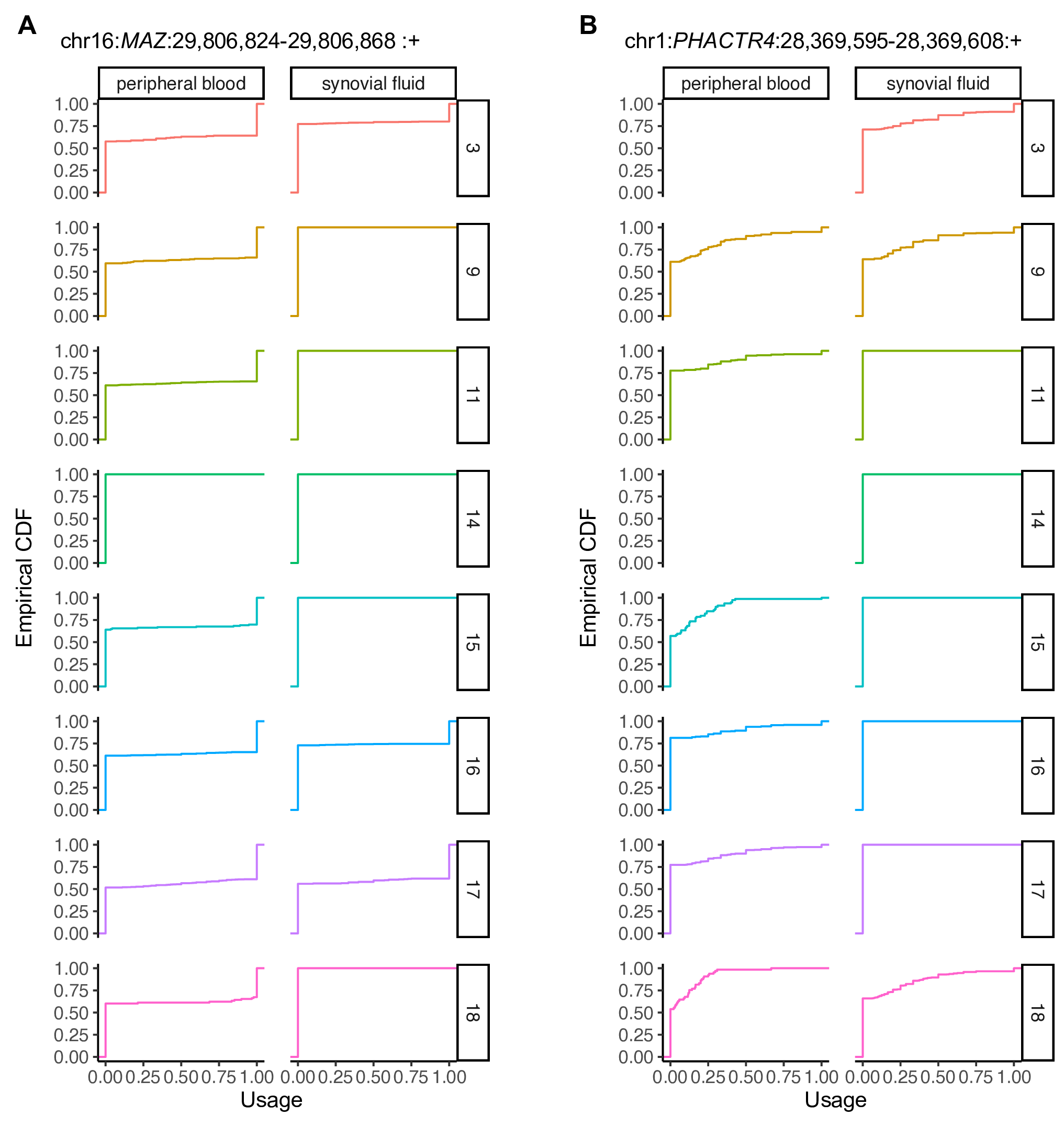

Supplement: S12 Fig — (A) MAZ. (B) PHACTR4. Each row of CDF curve represents the TSS usage distribution of one patient. If a gene was not detected in a patient, the corresponding panel would be empty. Both TSS clusters had higher usage in the PB samples. (TIF) [file pcbi.1012878.s012.tif]

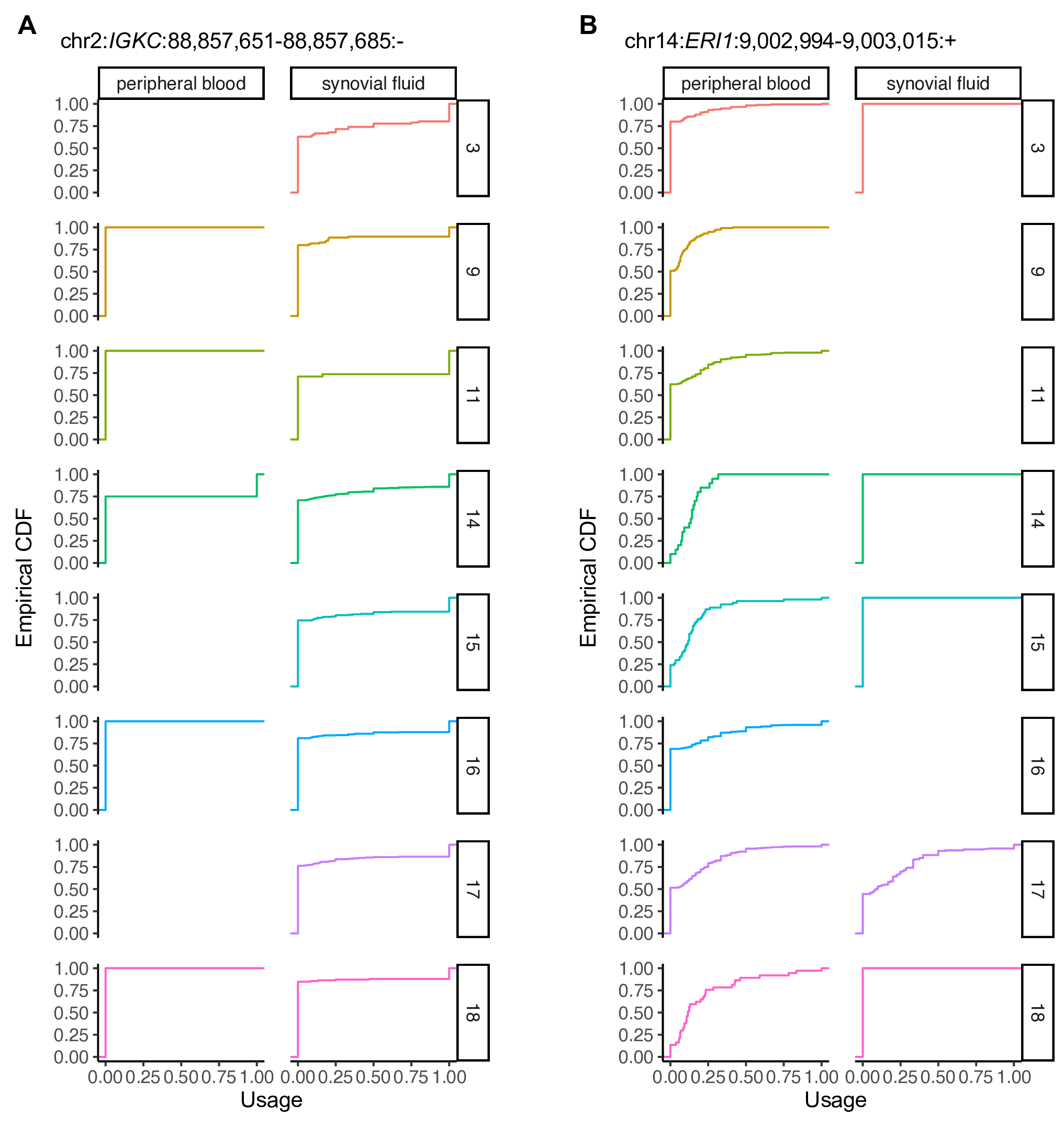

Supplement: S13 Fig — (A) IGKC. (B) ERI1. Each row of CDF curve represents the TSS usage distribution of one patient. If a gene was not detected in a patient, the corresponding panel would be empty. Both TSS clusters had higher usage in the PB samples. (TIF) [file pcbi.1012878.s013.tif]

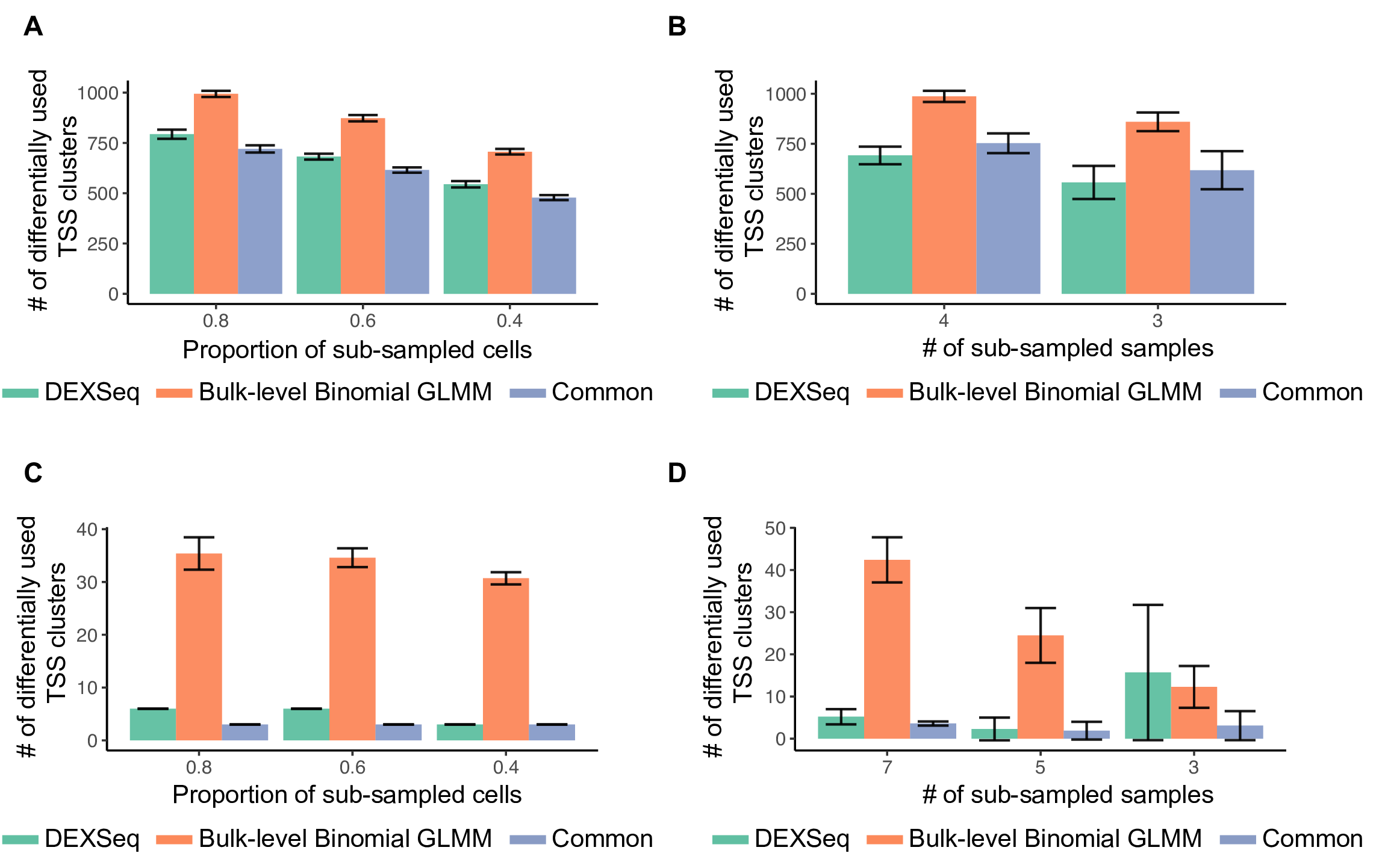

Supplement: S14 Fig — (A) Cell sub-sampling analysis of the COVID-19 dataset. The bars indicate the average number of differentially used TSS clusters identified by different methods across the 10 repeats. The error bars indicate the standard deviation of the numbers. (B) Sample sub-sampling analysis of the COVID-19 dataset. (C) Cell sub-sampling analysis of the Arthritis dataset. (D) Sample sub-sampling analysis of the Arthritis dataset. (TIF) [file pcbi.1012878.s014.tif]
